# Mapping the Nutritional Roles, Competency Domains, and Practice Gaps of Nephrology Nurses in Chronic Kidney Disease: A Scoping Review

**DOI:** 10.3390/healthcare14213798

**Published:** 2026-11-06

**Authors:** Gaetano Ferrara, Domenico Cannizzaro, Sofia Matteucci, Sara Morales Palomares, Anna Grimaldi, Elena Sandri, Giovanni Cangelosi, Marco Sguanci, Stefano Mancin, on behalf of the Italian Society of Nephrology Nurses (SIAN) Research Group

**Affiliations:** 1 Department of Biomedicine and Prevention, University of Rome Tor Vergata, Viale Montpellier 1, 00128 Rome, Italy; gaetano.ferrara@students.uniroma2.eu; 2 Italian Society of Nephrology Nurses (SIAN), Via Capotesta 1/30, 07026 Olbia, Italy; 3 Clinical and Experimental Medicine PhD Program, Department of Biomedical, Metabolic and Neural Sciences, University of Modena and Reggio Emilia, Via G. Campi 287, 41125 Modena, Italy; domenico.cannizzaro@unimore.it; 4 Scientific Institute for Research, Hospitalization and Healthcare (IRCCS), Humanitas Research Hospital, Via Manzoni 56, 20089 Rozzano, Italy; 5 Department of Pharmacy, Health and Nutritional Sciences (DFSSN), University of Calabria, 87036 Rende, Italy; 6 G-ALIS Research Group, Faculty of Medicine and Health Sciences, Catholic University of Valencia San Vicente Mártir, C/Quevedo, 2, 46001 Valencia, Spain; elena.sandri@ucv.es; 7 School of Pharmacy, Experimental Medicine and “Stefania Scuri” Public Health Unit, University of Camerino, Via Madonna Delle Carceri 9, 62032 Camerino, Italy

**Keywords:** chronic kidney disease, nephrology nurses, nutritional care, nursing roles, scoping review

## Abstract

**Highlights:**

**What are the main findings?**

- The literature suggests that Nephrology Nurses (NNs) contribute to nutritional education, adherence support, nutritional screening, and care coordination in adults with Chronic Kidney Disease (CKD).
- Five domains of nutritional nursing competence were identified: educational and communicative, clinical and advanced, collaborative and professional, general or basic nutritional, and guidance and counseling competencies.

**What are the implications of the main findings?**

- Structured competency frameworks and assessment approaches may help clarify and support the development of nutritional competencies among NNs.
- Targeted education and integrated care pathways may support nutritional risk identification and interdisciplinary care in CKD.

**Abstract:**

Background/Objectives: Chronic kidney disease (CKD) is frequently associated with malnutrition and protein-energy wasting. Nephrology nurses (NNs) contribute to nutritional education, counseling, screening, and monitoring, but their roles and competencies remain poorly defined. This scoping review mapped NNs’ nutritional roles and competencies in adult CKD care. Methods: This scoping review followed Joanna Briggs Institute (JBI) methodology and PRISMA-ScR reporting guidelines. The protocol was registered on the Open Science Framework (OSF) 10.17605/OSF.IO/YMW4F. The research question used the Population–Concept–Context framework: NNs as the population, nutritional nursing competencies as the concept, and adult nephrology care settings as the context. Eligible studies involved nurses in adult nephrology settings and addressed nutritional nursing roles, competencies, counseling, assessment tools, or related care processes. Searches were conducted on 23 May 2026 across PubMed/MEDLINE, Embase, CINAHL, Scopus, Web of Science, Cochrane Library, and Google Scholar. Two reviewers independently charted data using a structured form and synthesized findings into thematic domains. Results: Of 4699 identified records, 20 studies were included. Evidence encompassed nursing roles and activities, assessed or perceived competencies, knowledge gaps, and outcomes of nurse-led interventions, organized into five thematic domains: educational and communicative, clinical and advanced, collaborative and professional, general or basic nutritional, and guidance and counseling. The evidence was heterogeneous, predominantly derived from dialysis settings, and no formal critical appraisal was undertaken. Conclusions: NNs may contribute to nutritional CKD management, although the evidence does not uniformly reflect directly assessed competence. Structured training and standardized competency assessment may warrant further evaluation. No external funding was received.

## 1. Introduction

Chronic kidney disease (CKD) is a highly prevalent and clinically complex condition that represents a major global public health challenge [1]. In 2023, CKD affected an estimated 788 million adults aged 20 years and older worldwide, with a global age-standardized prevalence of 14.2%, and ranked as the ninth leading cause of death globally [2]. Beyond the progressive decline in kidney function, CKD is characterized by nutritional and metabolic alterations that require tailored management across disease stages, treatment modalities and care settings [3]. Malnutrition and protein-energy wasting (PEW) represent key nutritional concerns in CKD, particularly in older adults, for whom nutritional care requires careful balancing between the prevention of malnutrition and the management of CKD progression [4]. These conditions are multifactorial and may involve nutritional, inflammatory, metabolic and microbiota-related factors [5]. Evidence from non-dialysis CKD patients further supports the relevance of dietary energy and protein intake in relation to PEW [6]. In dialysis populations, malnutrition has been associated with increased mortality [7]. PEW has also been associated with CKD progression and adverse clinical outcomes [8], and has been described as a common complication among patients receiving maintenance hemodialysis [9]. Accordingly, nutritional care in CKD requires early assessment, ongoing monitoring and timely interventions across different nephrology care settings, particularly in light of the metabolic and nutritional consequences of kidney disease and kidney replacement therapy [3,10]. Given the complexity and longitudinal nature of nutritional care in CKD, nephrology nurses (NNs) are strategically positioned to contribute to its implementation across different care settings [11,12]. Nutrition is an integral component of CKD management, and kidney health professionals play a key role in supporting patients’ understanding of dietary interventions, which often require repeated adaptation according to disease progression and metabolic changes [11]. Within this context, NNs may contribute to nutritional education, counseling, screening, monitoring and reinforcement of dietary recommendations, while supporting patients in understanding and applying individualized dietary plans [11,13]. This role should be considered complementary to, rather than substitutive of, that of renal dietitians or other qualified nutrition professionals within multidisciplinary CKD care [3]. In adults with CKD, individualized medical nutrition therapy is provided by registered dietitian nutritionists or international equivalents, in collaboration with the multidisciplinary kidney care team [3]. Within this framework, NNs may contribute through nutritional screening, patient education, reinforcement of dietary recommendations, ongoing monitoring, adherence support, and referral to dietitians when specific nutritional needs or risks are identified, while also supporting the integration of nutritional recommendations into patients’ everyday self-management [11,14]. In dialysis care, structured dietary education has been shown to improve dietary attitudes and support patients in managing complex dietary recommendations, although changes in nutrient intake may be less consistent [13]. However, the contribution of NNs to nutritional care should be considered within the broader framework of nephrology nursing competencies. Recent scoping reviews have shown that NNs require a diverse set of core and advanced competencies to address the complexity of CKD care, including clinical practice, education, leadership, management and research skills [12]. At the same time, available evidence suggests that these competencies are acquired through heterogeneous pathways, combining structured education, mentorship, professional experience and individual learning, with a persistent lack of standardized postgraduate training in nephrology and dialysis nursing [15]. Despite increasing attention to nutritional care among NNs, nutritional competencies remain poorly defined and are often examined as separate dimensions rather than as a unified construct [12,15]. Studies have separately examined nutrition education and behavioral approaches in CKD, dietary counseling and recommendations in dialysis care, healthcare workers’ nutritional knowledge, attitudes and practices, the effects of nutrition education in hemodialysis patients, and broader competency and training frameworks in nephrology nursing [11,12,13,14,15,16,17]. This fragmentation is also reflected in recent instrument-development research, where nutritional competence in NNs has been operationalized as a multidimensional construct encompassing recommendations, attitudes, practice and advanced competencies (NECN-ESRD) [18]. A recent systematic review further synthesized the available evidence on nurses’ nutritional knowledge in hemodialysis care, reporting moderate levels of knowledge and identifying individual and professional factors, such as age, educational level and work experience, as possible determinants [19]. However, this review focused mainly on the cognitive dimension of competence and was limited to the hemodialysis setting. As a result, broader aspects of nutritional nursing competence, including patient education, dietary counseling, professional attitudes, interprofessional collaboration, training needs and the use of nutritional assessment tools across nephrology care settings, remain insufficiently mapped. This fragmentation highlights the need for a broader synthesis of the nutritional role and competencies of NNs across CKD care settings. Such a synthesis may provide a clearer understanding of the current evidence base and help inform future competency development, education, and research in NNs.

### Aims and Research Questions

The main aim of this scoping review is to map the nutritional role of NNs in the care of adults with CKD, with particular attention to nutritional competency domains and gaps in clinical practice.

Specifically, this review aims to:

- Map and describe the roles, activities, and responsibilities of NNs in the nutritional care of adults with CKD;
- Identify and characterize the domains of nutritional competencies described or attributed to NNs in CKD care;
- Examine how nutritional roles and competencies are described and operationalized across different nephrology care settings;
- Identify gaps in clinical practice related to the nutritional role and competencies of NNs;
- Identify gaps in education and competency development related to the provision of nutritional care by NNs.

Research Questions

The main research question is the following:

What is known about the nutritional role of NNs in the care of adults with CKD?

The following sub-questions will be addressed:

- What roles, activities, and responsibilities do NNs undertake in the nutritional care of adults with CKD?
- What domains of nutritional competency are described or attributed to NNs in CKD?
- How are nutritional roles and competencies described and operationalized across different nephrology care settings?
- What gaps in clinical practice are reported regarding the nutritional role and competencies of NNs?
- What gaps in education and competency development are identified in relation to the nutritional role of NNs?

## 2. Materials and Methods

### 2.1. Protocol Registration and Reporting

This scoping review was conducted following a predefined protocol prospectively registered on the Open Science Framework (OSF) (registration DOI: https://doi.org/10.17605/OSF.IO/YMW4F) and developed in accordance with the methodological guidance of the Joanna Briggs Institute (JBI) for scoping reviews [20,21]. To ensure methodological transparency and rigor, the review adhered to the Preferred Reporting Items for Systematic Reviews and Meta-Analyses extension for Scoping Reviews (PRISMA-ScR) for reporting [22] (Checklist in the Supplementary File S1). Compared with the registered protocol, the Population, Concept, Context (PCC) framework, population was clarified as NNs rather than adults with CKD, who represent the recipients of care. Trial protocols, trial registry records, and non-peer-reviewed sources were excluded from the final synthesis, while the planned narrative synthesis was refined into five inductively derived thematic domains.

### 2.2. Formulation of the Research Question

The research question was formulated according to the PPC framework, as recommended by JBI [20]. This framework provides a structured approach for defining the scope of a scoping review. Accordingly, the PCC elements were defined as follows: *Population* (**P**): NNs; *Concept* (**C**): nutritional nursing competencies, including knowledge, skills, attitudes, educational roles, counseling activities, and assessment tools; *Context* (**C**): nephrology settings, including hospital nephrology units, dialysis units, and outpatient CKD care settings.

For the purposes of this review, NNs were identified based on their documented employment in nephrology or renal care settings, including dialysis units and outpatient CKD services, or their explicitly described renal-specific nursing responsibilities. These activities included nutritional screening, monitoring, patient education, counseling, adherence support, and referral when reported. Formal specialization in NNs was not required. In studies involving mixed nursing populations, professional roles and clinical settings were distinguished according to the information provided in the original studies.

### 2.3. Eligibility Criteria

The inclusion criteria for this study were as follows: (1) studies involving nurses working in nephrology settings providing care to adult patients with CKD or end-stage renal disease (ESRD); (2) studies describing nutritional nursing competencies, counseling activities, or the use of nutritional assessment tools; (3) studies reporting clinical activities, care processes, educational interventions, or practice-related elements relevant to nutritional nursing competencies; and (4) documents from scientific societies or peer-reviewed literature illustrating educational aspects or professional roles related to nutritional care in nephrology. No language restrictions were applied in order to maximize the comprehensiveness of the review and reduce potential language bias. No restrictions were applied regarding the year of publication, in order to provide a broad mapping of the available evidence. The exclusion criteria were: (1) studies focusing on pediatric populations; (2) studies conducted exclusively in post-kidney transplant follow-up settings; (3) studies evaluating nutritional or educational interventions primarily directed at patients, when the nursing role or nutritional nursing competencies were not clearly described or analyzed; and (4) studies conducted outside nephrology settings. Editorials, commentaries, letters, book chapters, conference abstracts, trial protocols, trial registry records, and articles not available in full text were also excluded from the analysis. These publication types were excluded because they did not provide sufficiently complete and peer-reviewed evidence for the purposes of the present synthesis. Studies involving mixed nursing or renal patient populations were assessed according to the presence of identifiable components relevant to nutritional nursing care in CKD. Nurses from other clinical departments or with unspecified nephrology affiliation were not automatically classified as NNs. Where available, subgroup-specific findings were distinguished from those reported for broader populations. Otherwise, findings from mixed samples, including patients with acute kidney injury (AKI) or post-transplant status, were not attributed exclusively to NNs or patients with CKD. CKD-specific educational evidence from nurses with unspecified nephrology affiliation was interpreted as indirect evidence rather than as a direct assessment of NNs skills.

### 2.4. Search Strategy

The literature search was conducted on 23 May 2026 across six electronic databases: PubMed/MEDLINE via PubMed, Embase via Embase.com (Elsevier), CINAHL via EBSCOhost, Scopus via Elsevier, Web of Science via Clarivate, and the Cochrane Library, with coverage from database inception and no publication-date limits applied. The search strategy was developed using a combination of controlled vocabulary terms (e.g., MeSH and Emtree) and free-text keywords related to NNs and chronic kidney disease, nursing competencies and professional roles, and nutrition and nutritional care, connected through Boolean operators (AND, OR), and was tailored to meet the eligibility criteria of this review. No filters or limits were applied during the database searches; the predefined inclusion and exclusion criteria described in Section 2.3 were applied during the screening process. In addition to database searching, gray literature was explored using Google Scholar on 23 May 2026. All records retrieved from Google Scholar in the default relevance order were imported into Rayyan and screened by title and abstract. No stopping threshold was applied. All potentially relevant records retrieved from the searches were imported into Rayyan (free version; Rayyan Systems Inc., Cambridge, MA, USA), where duplicate records were automatically removed [23]. Title and abstract screening was performed independently by two reviewers (G.F. and S.M.) using Rayyan’s blind screening function. Records were screened using the bibliographic information available in Rayyan including English-language titles and abstracts where available, without excluding records solely on the basis of language. No translations were required during title and abstract screening. Subsequently, the full texts of potentially eligible reports were independently assessed by the same two reviewers against the predefined eligibility criteria described in Section 2.3. Disagreements arising at either screening stage were resolved through discussion and consensus, with the involvement of a third reviewer (M.S.) when necessary. All reports assessed for full-text eligibility were available in English; therefore, no full-text translation was required. Finally, in accordance with the JBI methodological framework [20], the reference lists of included articles were manually screened to identify additional eligible studies. A detailed search strategy for each database, including all controlled vocabulary terms, free-text keywords, and Boolean operators based on the original search documentation available to the authors, is provided in Supplementary File S2.

### 2.5. Data Extraction Process

In order to collect and organize the information required to answer the research question and achieve the objectives of this scoping review, a structured data extraction form was developed in accordance with the methodological recommendations of the JBI [20]. No formal pilot testing or calibration of the data extraction table was conducted. Data were extracted directly into the structured table developed for this review. The data extraction was performed independently by two reviewers (G.F. and S.M.), and any discrepancies were resolved through discussion. When consensus could not be reached, a third reviewer (M.S.) was consulted to ensure methodological rigor. For each included study, key information was systematically extracted and recorded. This included the authors’ names, year of publication, and country of origin, as well as the study design and the clinical setting in which the research was conducted. Additional details were collected on the characteristics of the study population, the main objectives and outcomes, and the principal findings related to nutritional nursing competencies. Particular attention was given to the nature of the evidence reported in each study, including nursing roles and activities, measured or perceived competencies, knowledge gaps or training needs, and patient- or process-related outcomes of nurse-led nutritional interventions. No study authors or investigators were contacted to obtain additional or confirmatory data. No data were imputed; information not reported in the original sources was recorded as not reported. For synthesis purposes, conceptually related findings were grouped according to the thematic classification described in Section 2.6. The complete data extraction matrix for all included studies is provided in Supplementary File S3.

### 2.6. Results Synthesis

The synthesis of the findings was conducted using a narrative approach, which allowed for a comprehensive description and interpretation of the evidence in relation to the main research questions. The evidence was further structured around major conceptual domains, including clinical practice implications, educational and training aspects, nutritional care processes, and the use of assessment tools in nutritional care. A specific section was dedicated to the analysis of educational pathways and training programs aimed at developing or strengthening these competencies among NNs. To enhance clarity and support the narrative synthesis, tables and figures were used to summarize the characteristics of the included studies and highlight their main findings. To enhance conceptual clarity, the synthesis distinguished between different types of evidence, including nursing roles and activities, measured or perceived competencies, knowledge gaps and training needs, and patient- or process-related outcomes of nurse-led nutritional interventions. Patient outcomes following nurse-led interventions were interpreted as evidence of the implementation or potential effects of a nursing role, rather than as direct evidence of nurses’ competence unless competence itself was specifically assessed. The thematic domains were developed inductively from the findings extracted from the included studies. Extracted findings were examined and grouped according to conceptual similarity, allowing recurrent areas of nutritional nursing practice and competence to emerge from the evidence. To improve transparency and consistency in domain assignment, operational boundaries were defined according to the primary function of each extracted finding. Educational and communicative findings primarily concerned the provision and structured communication of nutritional information. Guidance and counseling findings were characterized by individualized and interactive support, including tailored dietary guidance, negotiation, adherence support, and reinforcement of recommendations. Clinical and advanced findings primarily concerned nutritional risk identification, structured assessment, biochemical or anthropometric monitoring, care planning, and referral to specialist nutritional care. Each study was assigned to a primary domain according to its main objective and predominant focus. When findings could fit more than one domain, assignment was based on their predominant function and context in the original study; a secondary domain was retained only when a substantial and conceptually distinct finding supported it. Disagreements in domain assignment were resolved through discussion among the reviewers, with consultation of a third reviewer when necessary. Across these different types of evidence, findings were then organized into five thematic domains of nutritional nursing practice and competence. These domains reflect the multifaceted nature of the nursing role in the nutritional management of patients with CKD. The educational and communicative domain (1) encompasses the nurse’s ability to deliver nutritional education, structured teaching, and effective communication of nutritional information. The clinical and advanced domain (2) relates to competencies in nutritional screening, biochemical and anthropometric monitoring, individualized care planning, and evidence-based clinical decision-making. The collaborative and professional domain (3) emphasizes teamwork, interdisciplinary collaboration, and engagement with both patients and their families in shared care models. The general or basic nutritional domain (4) refers to fundamental knowledge of renal nutrition, phosphorus management, and professional attitudes toward nutritional care in CKD. Finally, the guidance and counseling domain (5) encompasses individualized dietary guidance, adherence support, negotiation, and tailoring or reinforcement of recommendations according to patients’ needs and barriers. Accordingly, assignment to a thematic domain did not imply that competence had been directly measured or demonstrated in every study within that domain. No formal stakeholder consultation was conducted, as this step was not included in the prospectively registered review protocol, which focused on mapping and synthesizing the available published evidence. The identified thematic domains were therefore derived from the included literature through the predefined data extraction and narrative synthesis process.

## 3. Results

**Figure 1:**
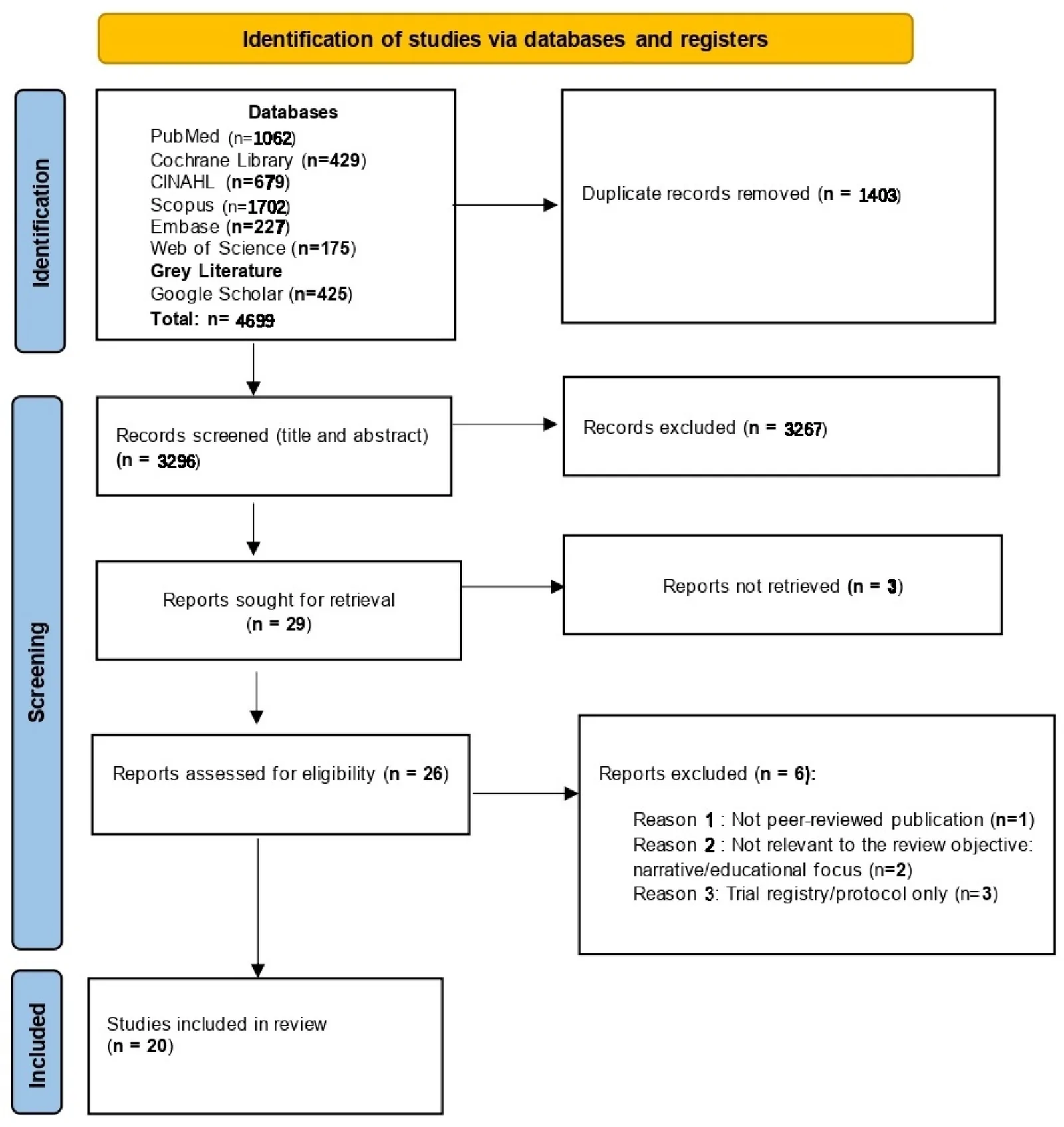
PRISMA Flow Chart.

A total of 4699 records were identified through the adopted search strategy. After the removal of 1403 duplicate records, 3296 records remained for title and abstract screening. Of these, 3267 records were excluded during title and abstract screening because they did not meet the predefined eligibility criteria. These mainly included studies not involving NNs, studies conducted outside nephrology settings or in non-CKD populations, and studies addressing nutritional or educational interventions without a clearly described nursing role or nutritional nursing competency. Full texts were sought for the remaining 29 reports, of which three could not be retrieved. The remaining 26 reports underwent full-text eligibility assessment, and six were excluded for the following reasons: not peer-reviewed publication (n = 1), narrative or educational focus not meeting the eligibility criteria (n = 2), and trial protocol or trial registry record (n = 3). Finally, 20 studies met the eligibility criteria and were included in the final review [14,18,24,25,26,27,28,29,30,31,32,33,34,35,36,37,38,39,40,41]. The study selection process is presented in Figure 1.

### 3.1. General Characteristics of the Studies Included

**Figure 2:**
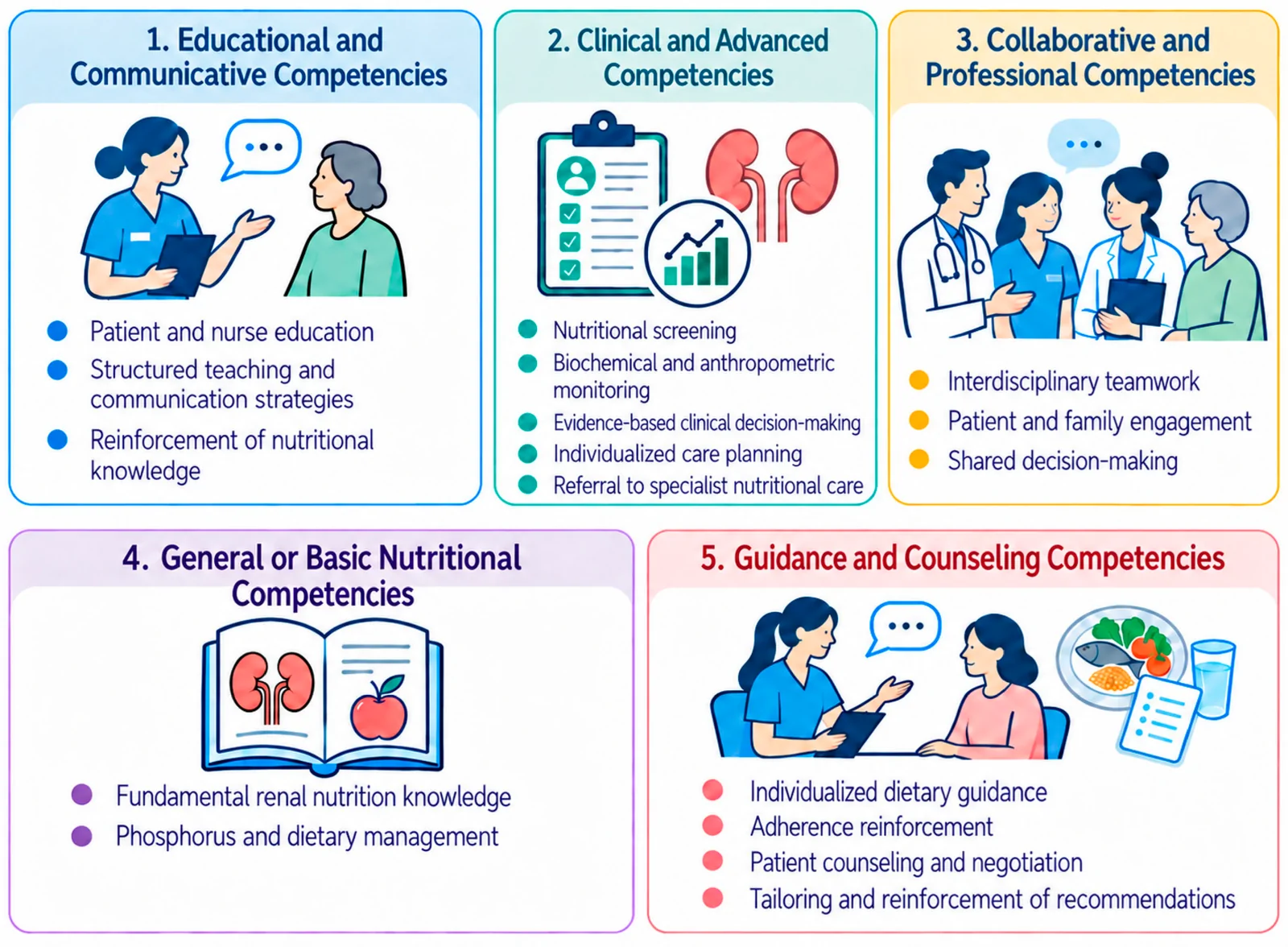
Visual summary of the five thematic domains of nutritional nursing competence among NNs.

The included articles were published between 2000 and 2026 [14,18,24,25,26,27,28,29,30,31,32,33,34,35,36,37,38,39,40,41]. Sample sizes varied widely across the included studies, ranging from n = 8 to n = 405. This variability reflected the heterogeneity of study designs and target populations. The geographical distribution included studies conducted in Asia (Iran, Iraq, India, China, Indonesia, Brunei Darussalam, and Turkey) [24,25,26,27,28,29,30,31,32,40,41], Europe (Italy and Greece) [18,33,34,35,36], Oceania (Australia and New Zealand) [14,37,38], and Africa (Morocco) [39]. The included studies comprised four randomized controlled trials (RCTs) [14,24,30,32], five quasi-experimental, nonrandomized, or before–after intervention studies [25,33,37,39,40], seven cross-sectional studies [26,27,29,34,35,36,41], two qualitative studies [28,31], one prospective validation study [38] and one methodological and cross-sectional psychometric validation study [18]. Overall, the included studies predominantly employed quantitative and interventional methodologies, complemented by qualitative approaches exploring the experiential and communicative dimensions of NNs. The general characteristics and main findings of the included studies are summarized in Table 1, while Table 2 presents the five thematic domains of nutritional nursing competence, their key competency areas, and their implications for practice. The complete data extraction matrix is provided in Supplementary File S3. Figure 2 visually summarizes the five thematic domains of nutritional nursing competence and their main competency areas.

### 3.2. Thematic Domains of Nutritional Nursing Competence in Nephrology

The included studies [14,18,24,25,26,27,28,29,30,31,32,33,34,35,36,37,38,39,40,41] provided heterogeneous forms of evidence regarding nutritional nursing practice. Some studies assessed nurses’ nutritional knowledge, others examined self-reported competencies, while others described nursing roles and activities or evaluated patient- and process-related outcomes following nurse-led interventions. Therefore, the five domains presented below should be interpreted as thematic areas of nutritional nursing practice and competence, rather than as domains in which competence was directly demonstrated in every included study.

#### 3.2.1. Educational and Communicative Competencies

The educational and communicative domain was the most frequently represented across the included studies. Six studies addressed educational interventions relevant to CKD nutritional care: five examined nurse-led patient education [24,30,37,39,40], whereas Fernandes et al. [25] evaluated a structured teaching program aimed at improving hospital staff nurses’ CKD-specific nutritional knowledge. These studies therefore provided distinct forms of evidence, including patient-related outcomes of nurse-led education and directly assessed nutritional knowledge among nurses receiving training.

Several interventional studies showed that structured nurse-led education may improve patient adherence and selected clinical or biochemical outcomes. Baraz et al. [24] evaluated oral and video-based educational interventions in hemodialysis patients and found that dietary and fluid compliance, assessed through biochemical parameters and interdialytic weight gain, was observed in 63.5% and 76.2% of patients in the oral and video education groups, respectively, with no significant difference between the two educational strategies. Similarly, Shi et al. [30] found that a nurse-led intensive educational program for patients with hyperphosphatemia significantly improved phosphate-related knowledge and contributed to reductions in serum phosphorus and calcium–phosphorus product levels, with effects sustained during follow-up. Sandlin et al. [37] also reported that a nurse-led educational intervention improved phosphate binder adherence and medication knowledge, although this improvement did not translate into significant changes in biochemical markers. More recently, Ouirdani et al. [39] evaluated a nurse-led educational intervention focused on dietary potassium management, fluid intake control, thirst management strategies, and basic physical activity recommendations in hemodialysis patients. The intervention was associated with a significant reduction in serum potassium levels and an increased proportion of patients within the recommended potassium range, although no significant changes were observed in quality of life or interdialytic weight gain. Şanlıtürk et al. [40] assessed a nurse-led animated video-based nutrition education program and found significant improvements in nutritional knowledge and fluid–diet adherence at 12 weeks, with additional exploratory improvements in interdialytic weight gain and selected biochemical parameters. This domain also included studies focused on nurses’ educational preparation and communicative experiences. Fernandes et al. [25] showed that a planned teaching program significantly improved staff nurses’ knowledge regarding the nutritional requirements of patients with CKD, supporting the value of structured educational interventions for improving nurses’ CKD-specific nutritional knowledge. Taken together, these studies highlight nurse-led patient education and the development of nurses’ own nutritional knowledge as distinct but complementary aspects of nutritional care in CKD.

#### 3.2.2. Clinical and Advanced Competencies

The clinical and advanced domain included five studies that addressed nutritional screening, structured assessment, biochemical and anthropometric monitoring, advanced decision-making, competency assessment, and the development of standardized tools for assessing nutritional nursing competencies in nephrology practice [14,18,35,38,41]. Compared with the educational domain, these studies focused more directly on the nurse’s role in identifying nutritional risk, monitoring clinical indicators, supporting evidence-based care pathways, and assessing competence in CKD dietary guidance. Bennett et al. [14] evaluated a monthly nurse-completed nutritional screening intervention in hemodialysis patients and found that dietetic referrals were approximately three times higher in the intervention group than in the control group, although no clinically significant changes were observed in quality of life, blood pressure, mortality, or most biochemical indices. This finding suggests that NNs may contribute to the early identification of patients requiring specialist nutritional support, particularly in dialysis settings where access to dietitians may be limited or intermittent. Xia et al. [38] developed and validated a renal-specific nutrition screening tool for hospitalized renal patients and demonstrated that the tool had high sensitivity and specificity when compared with the Subjective Global Assessment. Although nurse compliance with screening was low, the study supported the feasibility of nurse-led nutritional risk identification at ward level. In a national cross-sectional study among Italian NNs, Gazineo et al. [35] showed that nutritional competence remains an area of perceived weakness: 35% of nurses reported medium or low competence in nutrition, while 71% expressed interest in further educational pathways. Ferrara et al. [18] developed and validated the Nursing Education and Competencies in Nutritional care in patients with End Stage Renal Disease (NECN-ESRD) questionnaire among Italian NNs, providing a context-specific instrument to assess self-reported nutritional competencies in CKD and ESRD care. The study identified nutritional competence as a multidimensional construct composed of recommendations, attitudes, practice and advanced competencies, suggesting that standardized approaches to competency assessment, educational planning, and benchmarking in NNs’ practice may be useful and warrant further evaluation [18]. Similarly, Wang et al. [41] assessed clinical nurses’ self-reported competency in providing dietary guidance to patients with CKD using a validated assessment tool covering knowledge, attitudes/beliefs, and practices. The study reported a moderate overall competency score and identified departmental affiliation, dedicated learning time, prior nutrition training, training frequency, and leadership supervision as factors significantly associated with self-reported dietary guidance competency. Overall, the evidence within this domain suggests that clinical and advanced nutritional competencies extend beyond basic dietary advice. They include the ability to use screening tools, monitor biochemical and anthropometric data, recognize patients at nutritional risk, contribute to care planning, and apply standardized approaches to competency assessment. However, the available evidence also indicates that implementation may be limited by gaps in training, low screening compliance, and the absence of standardized competency frameworks, although validated instruments may support the assessment of nutritional nursing competencies in clinical and educational contexts.

#### 3.2.3. Collaborative and Professional Competencies

The collaborative and professional domain was represented by three studies that emphasized interdisciplinary care, family involvement, shared responsibility, and the integration of nutritional care within broader disease management models [32,33,35]. Although this domain was less frequently represented than the educational and clinical domains, it highlights an important dimension of NNs’ competence: the ability to coordinate nutritional care with patients, families, and other healthcare professionals. Wong et al. [32] evaluated a nurse-led disease management program for patients with CKD receiving continuous ambulatory peritoneal dialysis and found improvements in dietary non-adherence, CAPD non-adherence, symptom control, sleep, patient satisfaction, and selected quality-of-life outcomes. This study suggests that nutritional care may be more effective when embedded within multidimensional nursing interventions that address adherence, symptoms, education, and patient support together, rather than as isolated dietary counseling. Bordin et al. [33] focused on group educational meetings involving patients receiving peritoneal dialysis and their family members. The intervention addressed nutritional knowledge related to phosphorus, potassium, and water, while also recognizing the role of family members in food choices and meal preparation. This study suggests that nutritional education in peritoneal dialysis may benefit from extending beyond the individual patient and including the family context in which dietary choices and meal preparation often occur. Gazineo et al. [35] also contributed to this domain by highlighting the relevance of cross-cutting competencies, particularly communication, collaboration, decision-making, and professional development, within nephrology nursing practice. Together, these studies suggest that collaborative and professional competencies include the ability to engage families, work within multidisciplinary teams, coordinate care processes, and support shared decision-making. In NNs practice, nutritional care therefore appears not only as an individual educational activity, but also as a collaborative process requiring continuity, teamwork, and integration with the broader therapeutic plan.

#### 3.2.4. General or Basic Nutritional Competencies

The general or basic nutritional domain included five studies assessing foundational nutritional knowledge among NNs, particularly in relation to renal diet, phosphorus management, biochemical parameters, dietary assessment, and nutritional status in patients receiving hemodialysis or with renal failure [26,27,29,34,36]. These studies were mainly cross-sectional and consistently identified gaps in nurses’ nutritional knowledge. Cupisti et al. [34] assessed nutritional knowledge among hemodialysis patients, dialysis nurses, and controls, with a specific focus on phosphorus. Nurses obtained higher scores than patients and controls, but their knowledge of phosphorus was lower than their knowledge of other nutrients, indicating the need for further nutrition training even among dialysis staff. Similarly, Pafili et al. [36] found that renal nurses and hemodialysis patients had comparable levels of phosphorus nutritional knowledge, while nephrologists scored higher. The authors emphasized that continuing education for renal nurses and nephrologists could improve the nutritional care provided to hemodialysis patients. The Iraqi studies similarly showed variable but generally insufficient levels of nutritional knowledge among nurses. Rasheed et al. [29] reported that most nurses had a fair level of knowledge regarding the nutritional management of renal failure patients, with only a minority achieving good knowledge scores. Irzaij and Atiyah [27] assessed nurses’ knowledge toward nutritional status in patients with end-stage renal failure and highlighted the need to strengthen foundational nutritional knowledge in dialysis settings. Gaber et al. [26] found that most nurses working in hemodialysis centers had poor knowledge regarding biochemical parameters and dietary assessment, with age and years of experience significantly associated with knowledge levels. Overall, these studies suggest that basic nutritional competence remains an essential but unevenly developed component of NNs practice. Strengthening foundational knowledge through continuous and structured nutrition training may improve nurses’ ability to support dietary management and recognize nutrition-related risks in patients with CKD or renal failure.

#### 3.2.5. Guidance and Counseling Competencies

The guidance and counseling domain was represented by four studies addressing individualized dietary guidance, adherence support, patient negotiation, and reinforcement of recommendations [28,31,32,41]. This domain was distinguished from educational and communicative activities by its individualized and interactive nature, and from clinical and advanced activities by the absence of a primary focus on nutritional assessment, risk identification, clinical monitoring, or care planning. Emran and Zolkefli [28] highlighted the relational and individualized nature of dietary guidance, identifying strategies related to providing dietary information, encouraging patients to follow dietary plans, and negotiating with patients. Sulistyaningsih et al. [31] similarly emphasized intensive nurse–patient interaction, motivation, and ongoing education and counseling as strategies to support adherence to fluid and dietary restrictions. Wong et al. [32] showed that a nurse-led disease management model incorporating sustained follow-up and reinforcement of adherence behaviors was associated with improvements in dietary and treatment adherence. Finally, Wang et al. [41] assessed nurses’ self-reported competency in providing dietary guidance to patients with CKD, conceptualizing this competency through knowledge, attitudes/beliefs, and practice dimensions. Overall, these findings suggest that guidance and counseling competencies extend beyond the provision of nutritional information and involve individualized support, reinforcement of adherence, negotiation, and the practical application of dietary recommendations in everyday CKD care.

### 3.3. Practice and Educational Gaps

Across the included studies, recurrent practice and educational gaps emerged across multiple competency domains. Practice-related gaps included low compliance with nutritional screening [38] and perceived limitations in current approaches to dietary guidance [28]. Educational gaps included variable or insufficient nutritional knowledge and competence [26,27,29,34,35,36,41], the need for further structured education and professional development [25,27,31,34,35,41], and limited availability of standardized, context-specific approaches for assessing nutritional nursing competencies [18].

## 4. Discussion

This scoping review identified a multidimensional body of evidence concerning the nutritional roles and competencies of NNs caring for adults with CKD. The included studies differed substantially in the type of evidence provided, ranging from descriptions of nursing activities and nurse-led interventions to assessments of knowledge, perceived competence, and specific competency dimensions. The five thematic domains identified should therefore be interpreted as areas of nutritional nursing practice and competence rather than as uniformly demonstrated competencies, consistent with the broader literature on nephrology nursing competencies [12]. The findings also appear to indicate a possible gap between the nutritional responsibilities attributed to NNs and the extent to which these are formally defined and supported through professional education. In the Italian setting, evidence on perceived limitations in competence, participation in post-basic education, and interest in further training may support this interpretation [35]. This issue is particularly relevant in the context of contemporary CKD management, which requires early identification, risk stratification, timely intervention, and structured care models across the disease trajectory [42,43]. Within this framework, nutritional care specifically requires individualized assessment, ongoing monitoring, and multidisciplinary collaboration [44].

### 4.1. Educational and Communicative Competencies

The predominance of educational and communicative competencies suggests that nutritional education may represent an important component of NNs’ practice [24,25,30,37,39,40]. Some studies suggest potential benefits of structured education for nutritional knowledge, adherence, and selected clinical outcomes, although findings are not consistent [24,30,37,39,40]. This may indicate that, while relevant, education alone may not be sufficient to address the complexity of nutritional management in CKD [37,39]. These findings are consistent with the previous literature on educational interventions in CKD. A systematic review by Lopez-Vargas et al. found that effective educational interventions were commonly characterized by interactive, frequent, and multifaceted approaches, often incorporating goal setting, family involvement, and multidisciplinary participation [45]. Evidence from hemodialysis populations similarly supports the relevance of self-management and behaviorally informed approaches for improving adherence and selected clinical outcomes [46,47]. Moreover, dietary adherence is influenced by practical, communicative, and socioeconomic barriers, reinforcing the need to tailor nutritional education to the individual context of the patient rather than relying on standardized information alone [48]. From this perspective, educational competence in nephrology nursing can be conceptualized as the capacity to help patients translate complex nutritional recommendations into sustainable self-management behaviors over time. This interpretation is consistent with chronic care models that emphasize proactive, longitudinal, and patient-centered management and the integration of education and self-management support within broader care pathways [49,50,51].

### 4.2. Clinical and Advanced Competencies

The evidence suggests that nutritional competence in NNs may extend beyond basic nutritional education to include risk identification, monitoring, and activation of appropriate care pathways [14,18,35,38,41]. Screening studies indicate a potential role for NNs in the early identification of nutritional problems [14,38], while also highlighting implementation challenges, including low compliance with screening tools [38]. This interpretation is consistent with the broader literature emphasizing that nutrition screening represents an important first step in identifying malnutrition risk in people with kidney disease and that kidney-specific approaches may be required because generic screening tools may not adequately capture the particular nutritional characteristics of this population [52]. However, the present review extends this evidence by showing that the availability of validated screening tools does not necessarily ensure their implementation in practice, as low compliance with screening was reported in one of the included studies [38]. A similar gap emerges when nutritional competence is considered from a professional perspective. Gazineo et al. reported that a relevant proportion of NNs perceived their nutritional competence as medium or low [35], whereas Wang et al. identified departmental affiliation, dedicated learning time, previous nutrition training, training frequency, and leadership supervision as factors associated with dietary guidance competency [41]. These findings suggest that advanced nutritional competence should not be considered solely as an individual attribute, but also as a capability that may be influenced by educational and organizational conditions. Structured approaches to competency assessment and care delivery may help clarify this area of practice. The NECN-ESRD instrument provides a multidimensional framework for assessing nutritional competence [18]. However, as it relies on self-reported competence, further research is needed to determine how such assessment approaches relate to observed clinical performance and patient-level outcomes. Overall, the evidence suggests that the clinical and advanced nutritional role of NNs depends not only on the availability of assessment tools, but also on the extent to which competencies are clearly defined and supported within clinical practice. This interpretation is consistent with the broader nephrology nursing literature, which distinguishes core and advanced competencies and highlights the need for more standardized competency development pathways [12], as well as with contemporary CKD guidance emphasizing structured, multidisciplinary, and patient-centered approaches to nutritional assessment and management [3,42].

### 4.3. Collaborative and Professional Competencies

The evidence suggests that nutritional competence in NNs may be embedded within multidisciplinary and patient-centered care processes, in which patient and family involvement may influence the application of dietary recommendations [32,33,35]. It also appears to rely on broader professional competencies, including communication, collaboration, decision-making, and professional development [35]. This interpretation is consistent with the literature emphasizing the contribution of nursing leadership and team-based practice to coordinated nephrology care [53,54]. More broadly, integrated models of CKD care emphasize the need for coordinated and system-wide approaches to disease management [55]. While the previous literature has primarily addressed nursing leadership, interdisciplinary teamwork, and integrated CKD care more broadly [53,54,55], the findings of the present review suggest that these collaborative processes may also be relevant to nutritional care, particularly through communication among patients, families, and members of the multidisciplinary team and the integration of nutritional recommendations within the overall care plan [32,33,35]. Overall, the available evidence suggests that collaborative and professional competencies complement educational and clinical competencies by supporting the coordination and continuity of nutritional care.

### 4.4. General or Basic Nutritional Competencies

The evidence suggests heterogeneous foundational nutritional knowledge among NNs, with possible gaps particularly in renal diet, phosphorus management, biochemical parameters, and nutritional assessment [26,27,29,34,36]. These findings are consistent with the previous literature reporting suboptimal or moderate nutritional knowledge among healthcare professionals involved in CKD care and hemodialysis nurses, together with limitations in renal nutrition education and training [16,19]. Similar patterns have also been observed beyond specialized dialysis settings, where nurses showed moderate CKD-related knowledge and a need for in-service education [56]. In particular, recurrent gaps in areas such as phosphorus management and dietary assessment [34,36] suggest that foundational nutritional knowledge represents an important basis for the educational, screening, monitoring, and counseling activities identified across the other competency domains. Educational interventions may therefore help strengthen this foundational domain. Nurse-focused programs have been associated with improvements in nutritional knowledge [25], and a systematic review of educational interventions for nurses caring for patients with CKD reported improvements in knowledge, skills competence, clinical decision-making, and nurse–patient interaction [57]. Contemporary renal nutrition guidance further requires the integration of nutritional assessment, dietary management, and ongoing monitoring across CKD care [3]. Overall, the available evidence suggests the value of structured and continuing educational approaches aimed at consolidating core nutritional knowledge and supporting greater consistency in nephrology nursing practice.

### 4.5. Guidance and Counseling Competencies

The findings suggest that guidance and counseling competencies may represent a distinct dimension of nutritional nursing practice, characterized by individualized dietary support, reinforcement of adherence, negotiation with patients, and the practical application of dietary recommendations [28,31,32,41]. This domain differs from educational and communicative competencies because it involves a more individualized and interactive process aimed at supporting patients in applying dietary recommendations in everyday life, rather than primarily providing or explaining nutritional information. This may be particularly relevant because adherence to nutritional recommendations can be influenced by multiple practical and contextual barriers. Previous qualitative research in hemodialysis populations has similarly identified time, convenience, and financial constraints as barriers to dietary adherence and highlighted the need for counseling approaches tailored to individual barriers and preferences [58]. This interpretation is also consistent with evidence showing that educational and self-management interventions can improve adherence to fluid, dietary, and medication-related recommendations in hemodialysis patients [59]. Moreover, intensive individualized nutritional counseling has been associated with improved adherence to dietary recommendations in patients with stage 3–4 CKD compared with a one-time group-based nutrition program, further supporting the potential value of tailored and sustained guidance in nutritional care [60]. Similarly, a randomized controlled trial protocol in peritoneal dialysis proposed a structured continuous nursing model incorporating nutritional education, individualized guidance and consultation, dietary self-monitoring, and longitudinal follow-up, illustrating how these activities could be integrated into a broader nursing care pathway [61]. However, NNs’ involvement in guidance and counseling should remain integrated within multidisciplinary care, as contemporary CKD guidance emphasizes individualized dietary management, the involvement of renal dietitians or accredited nutrition providers, and access to patient-centered multidisciplinary care incorporating dietary counseling and education [62]. Overall, these findings suggest that guidance and counseling competencies in NN practice may complement educational and communicative competencies by supporting the individualized application of nutritional recommendations, addressing barriers to adherence, and promoting sustained dietary self-management.

### 4.6. Strengths and Limitations

A major strength of this scoping review is its multidimensional mapping of nutritional nursing roles and competencies in CKD across diverse geographical, clinical, and methodological contexts. The synthesis organized the evidence into five domains—educational and communicative, clinical and advanced, collaborative and professional, general or basic nutritional, and guidance and counseling—while also incorporating evidence on educational interventions, clinical practices, and competency assessment. Several limitations should be acknowledged. The extraction form was not formally pilot-tested or calibrated, which may have affected the consistency and completeness of data charting across reviewers. The included studies were highly heterogeneous in design, sample size, populations, clinical settings, interventions, and outcome measures, limiting direct comparability. No formal critical appraisal or risk-of-bias assessment was undertaken; therefore, patient- and process-related outcomes should not be interpreted as direct evidence of nursing competence or intervention effectiveness. In addition, several studies relied on self-reported knowledge, attitudes, or competencies, which may be influenced by social desirability, recall bias, and inaccurate self-estimation. Most evidence also originated from dialysis settings, limiting transferability to earlier stages of CKD, where nutritional risk identification and self-management support may require different competencies. The geographical distribution of the evidence may also limit generalizability, as healthcare systems, organizational models, educational pathways, and access to nutrition professionals differ across settings. The absence of restrictions on publication year may also have introduced older evidence that is not fully aligned with contemporary nephrology practice. Although multiple databases and Google Scholar were searched, relevant studies may have been missed; Google Scholar results were screened in default relevance order, and reports unavailable in full text were excluded. The original search strategies included the truncation term “responsabil*” rather than “responsib*”, which may have reduced search sensitivity despite the inclusion of other relevant search terms. The absence of formal stakeholder consultation may limit the external validation and practice-based applicability of the identified domains. Finally, the five-domain framework should be regarded as a synthesis framework rather than a definitive taxonomy, as nutritional competencies are interconnected and frequently overlap in clinical practice. The available evidence also remains insufficient to determine which competencies should be considered essential or advanced across different nephrology settings or which educational pathways are most effective for their development. Overall, these gaps highlight the need for future studies using standardized and comparable competency assessment tools, with greater attention to pre-dialysis CKD and to outcomes linking NNs’ competency development with care processes and patients’ nutritional and clinical outcomes.

### 4.7. Implications for Practice

The findings of this scoping review suggest the need to move beyond a predominantly reactive and fragmented approach to nutritional care toward an integrated, continuous, personalized, and person-centered model [63]. Within this perspective, nutritional competency among NNs should not be regarded as an ancillary set of skills, but as a cross-cutting component of CKD management integrating nutritional risk assessment, therapeutic education, clinical monitoring, self-management support, and interdisciplinary coordination. Nutritional screening and education should therefore be embedded within routine care pathways and longitudinal follow-up, with particular attention to the early identification of nutritional needs and continuity of care [64]. A contemporary model of care should also move beyond the concept of nutritional education as the simple transmission of information. The educational and communicative competencies identified in this review support the development of personalized, interactive, and longitudinal approaches in which NNs facilitate self-management and behavioral change while adapting interventions to patients’ clinical characteristics, health literacy, dietary habits, and social and family contexts [65,66]. Accordingly, education for NNs may benefit from competency-based approaches aimed at explicitly defining and developing the nutritional competencies identified in this review, including clinical, communicative, collaborative, and counseling dimensions [67]. Beyond the evidence directly mapped in this review, digital technologies, artificial intelligence, and broader integrated-care perspectives may represent relevant areas for future research in NNs [68,69,70,71]. The emerging literature suggests that digital screening and monitoring tools, remote follow-up systems, interoperable health records, and Artificial Intelligence (AI)-supported decision-making may have potential applications in nutritional care, including risk identification, monitoring, patient education, and personalization of interventions [68,69,71]. However, these applications require appropriate clinical validation, professional oversight, system interoperability, data protection, and consideration of ethical and equity-related implications [70,72,73,74]. These considerations are prospective and should not be interpreted as findings derived from the studies included in this review. From an organizational and professional perspective, the contextual literature supports the development of structured competency pathways for NNs, encompassing foundational, advanced, and context-specific competencies according to levels of clinical complexity [75,76]. Validated competency assessment approaches may support the identification of educational needs, the design of targeted professional development programs, and the longitudinal monitoring of competency development [77]. Integrating competency assessment into clinical and educational governance may further support individualized professional development and the alignment of training with quality-of-care objectives. Overall, these contextual considerations support the development of more clearly defined nutritional competency pathways for NNs, integrating educational, clinical, communicative, collaborative, and counseling dimensions within multidisciplinary CKD care [78]. Future research should determine how these competencies can be effectively developed, assessed, and translated into clinical practice across different nephrology settings, while also exploring how digital health systems may support continuity and integration of care [79]. Such an approach may potentially support earlier identification of nutritional needs, more personalized care, and greater continuity across the CKD trajectory.

## 5. Conclusions

The available evidence suggests that NNs may contribute to nutritional management of CKD through educational and communicative activities, adherence support, nutritional screening and monitoring, and interdisciplinary collaboration. However, the available evidence encompasses assessed nutritional knowledge, self-reported competencies, nursing roles and activities, and patient- or process-related outcomes, rather than uniformly demonstrating nursing competence. Educational gaps persist, and evidence from earlier stages of CKD remains limited. Structured educational pathways and standardized competency assessment tools may be warranted and should be evaluated in future research. Additionally, the potential role of digital technologies and AI in nutritional care warrants further investigation, as these areas were not directly evaluated in the evidence mapped by this review.

## Figures and Tables

**Table 1:** Summary of the included studies.

Author, Year,	Country	Study Design	Sample Size	Study Objective	Main Findings	Nature of Evidence	Thematic Domain
Baraz et al., 2010 [24]	Iran	RCT	N = 63 HD patients	To assess the effect of nurse-led educational interventions on dietary and fluid compliance in hemodialysis patients	Both oral and video education improved dietary compliance and biochemical outcomes, especially in younger and more educated patients	Nursing role/intervention; patient outcomes	Educational and communicative
Bennett et al., 2013 [14]	Australia	RCT	N = 81 HD patients	To test whether monthly nurse-led nutrition screening increases dietitian referrals and affects outcomes	Nurse-led screening tripled dietitian referrals; no effect on mortality or quality of life	Nursing role/activity; process and patient outcomes	Clinical and advanced
Bordin et al., 2000 [33]	Italy	Quasi-experimental pre–post study (group education)	N = 22 CAPD patients + n = 17 family members	To assess nutritional knowledge and media influence after group educational sessions	Post-intervention errors decreased; group meetings improved knowledge and highlighted media influence	Nursing educational role/activity; knowledge outcomes	Collaborative and professional
Cupisti et al., 2012 [34]	Italy	Cross-sectional study	N = 191 patients, 105 nurses, 86 control group	To assess nutritional knowledge on phosphorus and other nutrients among patients and nurses	Phosphorus knowledge was lower than for other nutrients; nurses performed better but still had gaps; more nutrition training needed	Knowledge assessment; knowledge gap	General or basic nutritional
Fernandes et al., 2021 [25]	India	Uncontrolled before–after study	N = 40 hospital staff nurses (department not reported)	To evaluate the effect of a planned teaching program on nurses’ knowledge of CKD patients’ nutritional needs	Planned teaching significantly improved nurses’ nutritional knowledge (*p* < 0.001)	Knowledge assessment; training effect; indirect educational evidence	Educational and communicative
Ferrara et al., 2026 [18]	Italy	Methodological psychometric validation study	N = 405 nurses	To develop and validate the NECN-ESRD questionnaire for assessing nutritional competencies in nephrology nursing	The NECN-ESRD showed strong validity and reliability and identified four domains: Recommendations, Attitudes, Practice, and Advanced Competencies	Self-reported competency assessment; instrument validation	Clinical and advanced
Gaber, 2022 [26]	Iraq	Cross-sectional study	N = 80 HD nurses	To assess nurses’ knowledge on biochemical and dietary assessment in HD patients	Most nurses showed poor knowledge; significant differences by age and experience	Knowledge assessment; knowledge gap	General or basic nutritional
Gazineo et al., 2025 [35]	Italy	Cross-sectional study	N = 296 NNs	To assess basic and advanced competencies of nephrology nurses in Italy	35% reported medium/low nutritional competence; 71% interested in further training; strong communication and decision-making skills	Self-reported competency; training need	Clinical and advanced, collaborative and professional
Sulistyaningsih et al., 2021 [31]	Indonesia	Qualitative phenomenological study	N = 8 HD nurses	To explore nurses’ experiences in enhancing dietary and fluid adherence among HD patients	Five themes: intensive patient interaction, varied teaching methods, educational barriers, need for professional updating, and perceived benefits	Nursing role/experience; professional development need	Guidance and counseling
Wong et al., 2010 [32]	Hong Kong	RCT	N = 94 CAPD patients (47 intervention, 47 control)	To evaluate the effectiveness of a nurse-led disease management program on adherence and quality of life	Improved dietary and CAPD adherence, sleep quality, symptom control, and satisfaction; effects sustained at 13 weeks	Renal/general nurse-led intervention; patient outcomes	Collaborative and professional; guidance and counseling
Xia et al., 2016 [38]	New Zealand	Prospective validation study	N = 122 renal inpatients (63 HD, 30 PD, 19 non-dialysis CKD, 3 AKI, 5 post-transplant, 2 nephrotic syndrome)	To develop and validate the R-NST for early identification of undernutrition risk among renal inpatients	R-NST showed high accuracy vs. SGA and proved feasible for nurse-led nutritional screening despite low compliance	Nursing screening role/activity; tool validation and process implementation	Clinical and advanced
Rasheed et al., 2018 [29]	Iraq	Cross-sectional study	N = 51 HD nurses	To assess nurses’ knowledge of nutritional management in renal failure patients	Most nurses (70.6%) had fair knowledge; higher education was significantly associated with better knowledge	Knowledge assessment; knowledge gap	General or basic nutritional
Irzaij & Atiyah, 2021 [27]	Iraq	Cross-sectional study	N = 100 HD nurses	To assess nurses’ knowledge regarding nutritional status for ESRD patients	Most nurses had moderate knowledge; higher education significantly associated with better knowledge	Knowledge assessment; knowledge gap	General or basic nutritional
Pafili et al., 2019 [36]	Greece	Cross-sectional study	N = 68 HD patients, N = 19 renal nurses, N = 11 nephrologists	To assess phosphorus nutritional knowledge among dialysis patients and staff	Nurses and patients showed similarly low phosphorus knowledge; nephrologists scored higher but still lacked key diet knowledge	Knowledge assessment; knowledge gap	General or basic nutritional
Emran & Zolkefli, 2021 [28]	Brunei Darussalam	Qualitative descriptive study	N = 12 NNs	To explore nurses’ perceptions and experiences when giving dietary recommendations to dialysis patients	Three themes: information approaches, adherence promotion, and negotiation with patients; emphasized nurses’ educational and communicative role	Nursing role/experience; perceived practice gap	Guidance and counseling
Sandlin et al., 2013 [37]	Australia	Pre–post descriptive intervention study	N = 70 HD patients	To evaluate the impact of a nurse-led educational program on phosphate binder adherence and knowledge	Adherence improved from 44% to 72% (*p* = 0.016); knowledge increased; no significant change in biochemical markers	Nursing role/intervention; patient outcomes	Educational and communicative
Shi et al., 2013 [30]	China	Two-center RCT	N = 80 HD patients (40 intervention, 40 control)	To evaluate the effectiveness of a nurse-led intensive educational program on hyperphosphatemia management and phosphate knowledge	Significant reductions in serum phosphorus and Ca×P; improved knowledge of diet and phosphate binders; effects sustained for 6 months	Nursing role/intervention; patient outcomes	Educational and communicative
Ouirdani et al., 2026 [39]	Morocco	Quasi-experimental pre–post pilot study	N = 30 HD patients	To test a nurse-led education program for potassium control in HD patients	Improved serum potassium control; no significant effect on QoL or IDWG	Nursing role/intervention; patient outcomes	Educational and communicative
Şanlıtürk et al., 2026 [40]	Turkey	Nonrandomized pre–post controlled study	N = 107 HD patients	To assess nurse-led animated video nutrition education	Improved nutritional knowledge and fluid–diet adherence at 12 weeks	Nursing role/intervention; patient outcomes	Educational and communicative
Wang et al., 2026 [41]	China	Cross-sectional study	N = 393 clinical nurses (243 nephrology/blood purification; 150 other departments)	To assess nurses’ CKD dietary guidance competency	Self-reported competency was associated with department, training, learning time, and leadership support	Self-reported competency assessment; associated factors; mixed nursing sample	Clinical and advanced; guidance and counseling

**Legend**. HD: Hemodialysis; CAPD: Continuous Ambulatory Peritoneal Dialysis; PD: Peritoneal Dialysis; CKD: Chronic Kidney Disease; ESRD: End-Stage Renal Disease; RCT: Randomized Controlled Trial; SGA: Subjective Global Assessment; R-NST: Renal Nutrition Screening Tool; Ca×P: Calcium–Phosphorus Product; NECN-ESRD: Nursing Education and Competencies in Nutritional care in patients with ESRD; QoL: quality of life; IDWG: interdialytic weight gain; NNs: nephrology nurses; AKI: Acute Kidney Injury.

**Table 2:** Thematic domains of nutritional nursing competence.

Domain	Studies	Key Themes/Competency Focus	Implications for Practice
(1) Educational and Communicative Competencies	[24,25,30,37,39,40]	Patient and nurse education, structured teaching, communication strategies, reinforcement of nutritional knowledge	Enhanced patient engagement, improved adherence, selected biochemical outcomes
(2) Clinical and Advanced Competencies	[14,18,35,38,41]	Nutritional screening and risk identification, clinical monitoring, care planning, competency assessment, referral to specialist nutritional care	Early detection of malnutrition, evidence-based care, standardized competency assessment
(3) Collaborative and Professional Competencies	[32,33,35]	Interdisciplinary teamwork, patient-family collaboration, professional development	Improved care coordination, shared decision-making
(4) General or Basic Nutritional Competencies	[26,27,29,34,36]	Foundational renal nutrition knowledge, phosphorus and dietary management	Need for continuous training programs
(5) Guidance and Counseling Competencies	[28,31,32,41]	Individualized dietary guidance, counseling, adherence support, negotiation, tailoring and reinforcement of recommendations	Improved adherence, patient self-management, and individualized support

Note. The thematic domains synthesize different types of evidence, including assessed nutritional knowledge, self-reported competencies, nursing roles and activities, and patient- or process-related outcomes. Domain allocation does not imply that nursing competence was directly assessed or demonstrated in every included study.

## Data Availability

All data supporting the findings of this review are available within the article and its Supplementary Materials.

## Supplementary Materials

The following supporting information can be downloaded at: https://www.mdpi.com/article/10.3390/healthcare14213798/s1, File S1: PRISMA-ScR checklist; File S2: Search strategies for each database; File S3: Complete data extraction matrix of included studies; File S4: PRISMA 2020 for Abstracts Checklist.

## Abbreviations

The following abbreviations are used in this manuscript:

AKI	Acute Kidney Injury
CAPD	Continuous Ambulatory Peritoneal Dialysis
Ca×P	Calcium–Phosphorus Product
CCM	Chronic Care Model
CKD	Chronic Kidney Disease
ESRD	End-Stage Renal Disease
HD	Hemodialysis
JBI	Joanna Briggs Institute
NECN-ESRD	Nursing Education and Competencies in Nutritional Care in Patients with End-Stage Renal Disease
OSF	Open Science Framework
PCC	Population–Concept–Context
PD	Peritoneal Dialysis
PEW	Protein-Energy Wasting
PRISMA-ScR	Preferred Reporting Items for Systematic Reviews and Meta-Analyses Extension for Scoping Reviews
RCT	Randomized Controlled Trial
R-NST	Renal Nutrition Screening Tool
RRT	Renal Replacement Therapy
SGA	Subjective Global Assessment
NNs	Nephrology Nurses

## Appendix A

Italian Society of Nephrology Nurses (SIAN) Board of Directors (contributed as authors of the manuscript):

1. Domenica Gazineo: Italian Society of Nephrology Nurses (SIAN) Board of Directors, Via Capotesta 1/30, 07026 Olbia, Italy; Governo Clinico e Qualità, IRCCS Azienda, Ospedaliero-Universitaria di Bologna, Bologna, Italy; domenica.gazineo3@unibo.it;
2. Addolorata Palmisano: Italian Society of Nephrology Nurses (SIAN) Board of Directors, Via Capotesta 1/30, 07026 Olbia, Italy; dorianapalmisano77@gmail.com;
3. Alessandro Pizzo: Italian Society of Nephrology Nurses (SIAN) Board of Directors, Via Capotesta 1/30, 07026 Olbia, Italy; pizzoalexxandro@gmail.com;
4. Silvia Cappelletti: Italian Society of Nephrology Nurses (SIAN) Board of Directors, Via Capotesta 1/30, 07026 Olbia, Italy; silviacappelletti74@outlook.com;
5. Angela Greco: Italian Society of Nephrology Nurses (SIAN) Board of Directors, Via Capotesta 1/30, 07026 Olbia, Italy; angelagreco1967@gmail.com;
6. Francesco Barci: Italian Society of Nephrology Nurses (SIAN) Board of Directors, Via Capotesta 1/30, 07026 Olbia, Italy; francescobarci81@gmail.com.

## References

[1] (2024). Kidney Disease: Improving Global Outcomes (KDIGO) CKD Work Group. KDIGO 2024 clinical practice guideline for the evaluation and management of chronic kidney disease. Kidney Int..

[2] GBD 2023 Chronic Kidney Disease Collaborators (2025). Global, regional, and national burden of chronic kidney disease in adults, 1990–2023, and its attributable risk factors: A systematic analysis for the Global Burden of Disease Study 2023. Lancet.

[3] Ikizler T.A., Burrowes J.D., Byham-Gray L.D., Campbell K.L., Carrero J.J., Chan W., Fouque D., Friedman A.N., Ghaddar S., Goldstein-Fuchs D. (2020). KDOQI clinical practice guideline for nutrition in CKD: 2020 update. Am. J. Kidney Dis..

[4] Piccoli G.B., Cederholm T., Avesani C.M., Bakker S.J.L., Bellizzi V., Cuerda C., Cupisti A., Sabatino A., Schneider S., Torreggiani M. (2023). Nutritional status and the risk of malnutrition in older adults with chronic kidney disease—Implications for low protein intake and nutritional care: A critical review endorsed by ERN-ERA and ESPEN. Clin. Nutr..

[5] Martín-Del-Campo F., Avesani C.M., Stenvinkel P., Lindholm B., Cueto-Manzano A.M., Cortés-Sanabria L. (2023). Gut microbiota disturbances and protein-energy wasting in chronic kidney disease: A narrative review. J. Nephrol..

[6] Han Q., Zhang R., Wu J., He F., Qing F., Li W., Chen C., Yang Q. (2024). Cross-sectional relationship between dietary protein intake, energy intake and protein energy wasting in chronic kidney disease patients. Br. J. Nutr..

[7] Rashid I., Sahu G., Tiwari P., Willis C., Asche C.V., Bagga T.K., Ghule P., Bland A. (2024). Malnutrition as a potential predictor of mortality in chronic kidney disease patients on dialysis: A systematic review and meta-analysis. Clin. Nutr..

[8] Narasaki Y., Rhee C.M., Kalantar-Zadeh K., Rastegar M. (2025). Why protein-energy wasting leads to faster progression of chronic kidney disease. Curr. Opin. Nephrol. Hypertens..

[9] Zhang J., Xu F., Qing W., Zhuang P., Xu X. (2025). Research progress on influencing factors and intervention methods of protein-energy wasting in maintenance hemodialysis patients: A literature review. Medicine.

[10] Fiaccadori E., Sabatino A., Barazzoni R., Carrero J.J., Cupisti A., De Waele E., Jonckheer J., Singer P., Cuerda C. (2021). ESPEN guideline on clinical nutrition in hospitalized patients with acute or chronic kidney disease. Clin. Nutr..

[11] Khor B.H., Sumida K., Scholes-Robertson N., Chan M., Lambert K., Kramer H., Lui S.F., Wang A.Y.M. (2023). Nutrition education models for patients with chronic kidney disease. Semin. Nephrol..

[12] Andreoli D., Morales Palomares S., Mancin S., Parozzi M., Gazineo D., Palmisano A., Angileri S.A., Ricco M., Anastasi G., Savini S. (2025). Exploring the competencies of nephrology nurses: A comprehensive scoping review. Int. Nurs. Rev..

[13] Akeren Z., Semerci Çakmak V., Kalyoncuo S. (2025). The effect of dietary education on attitudes and behaviors in hemodialysis patients: A randomized trial. Contemp. Clin. Trials.

[14] Bennett P.N., Miller M.D., Woodman R.J., Hill K., Murray S., Gleadle J.M. (2013). Nutrition screening by nurses in dialysis. J. Clin. Nurs..

[15] Parozzi M., Andreoli D., Morales Palomares S., Cappelletti S., Anastasi G., Godino L., Gazineo D., Ferrara G., Sguanci M., Mancin S. (2025). Exploring educational and training skills of nephrology nurses: Scoping review. G. Ital. Nefrol..

[16] Munuo A.E., Mugendi B.W., Kisanga O.A., Otieno G.O. (2016). Nutrition knowledge, attitudes and practices among healthcare workers in management of chronic kidney diseases in selected hospitals in Dar es Salaam, Tanzania: A cross-sectional study. BMC Nutr..

[17] Ouirdani M., Boutib A., Azizi A., Chergaoui S., Saad E.M., Hilali A., Marfak A., Youlyouz-Marfak I. (2024). Impact of nutrition education on various health-related components of hemodialysis patients: A systematic review. Healthcare.

[18] Ferrara G., Bozzetti M., Sguanci M., Bonetti L., Morales Palomares S., Sandri E., Cangelosi G., Napolitano D., Mancin S., Piredda M. (2026). Questionnaire on nursing competencies in nutritional care for chronic kidney patients: Development and validation. Nurs. Rep..

[19] Alizadeh T., Karkhah M.R., Arasteh M., McFarlane P., Okamoto Y., Sandanasamy S., Takasi P. (2024). Nurses’ knowledge and related factors towards hemodialysis patients’ nutrition: A systematic review. J. Nurs. Rep. Clin. Pract..

[20] Peters M.D.J., Marnie C., Tricco A.C., Pollock D., Munn Z., Alexander L., McInerney P., Godfrey C.M., Khalil H. (2020). Updated methodological guidance for the conduct of scoping reviews. JBI Evid. Synth..

[21] Pollock D., Tricco A.C., Peters M.D.J., McInerney P.A., Khalil H., Godfrey C.M., Alexander L., Munn Z. (2022). Methodological quality, guidance, and tools in scoping reviews: A scoping review protocol. JBI Evid. Synth..

[22] Tricco A.C., Lillie E., Zarin W., O’Brien K.K., Colquhoun H., Levac D., Moher D., Peters M.D.J., Horsley T., Weeks L. (2018). PRISMA extension for scoping reviews (PRISMA-ScR): Checklist and explanation. Ann. Intern. Med..

[23] Ouzzani M., Hammady H., Fedorowicz Z., Elmagarmid A. (2016). Rayyan—A web and mobile app for systematic reviews. Syst. Rev..

[24] Baraz S., Parvardeh S., Mohammadi E., Broumand B. (2010). Dietary and fluid compliance: An educational intervention for patients having haemodialysis. J. Adv. Nurs..

[25] Fernandes S.T., Dsilva F., Dsouza S.M. (2021). Effectiveness of a planned teaching program on the knowledge of staff nurses with regard to the nutritional requirements of patients with chronic kidney disease. J. Health Allied Sci. NU.

[26] Gaber A.H., Al-Ashour I.A. (2022). Nurses’ knowledge regarding biochemical parameter and dietary assessment for patient undergoing hemodialysis. J. Posit. Sch. Psychol..

[27] Irzaij I.A., Atiyah H.H. (2021). Nurses’ knowledge toward nutritional status for patients with end stage renal failure at Al-Hussain Teaching Hospital in Al-Nasiriyah City. Indian J. Forensic Med. Toxicol..

[28] Emran S.N.H., Zolkefli Y. (2021). Nephrology nurses’ views in giving a dietary recommendation for dialysis patients: An interview study. Belitung Nurs. J..

[29] Rasheed H.A., Aziz N.G., Osman G.A., Younis Y.M. (2018). Nurses’ knowledge of the nutritional management of renal failure in Erbil City, Kurdistan Region, Iraq. Zanco J. Med. Sci..

[30] Shi Y.X., Fan X.Y., Han H.J., Wu Q.X., Di H.J., Hou Y.H., Zhao Y. (2013). Effectiveness of a nurse-led intensive educational programme on chronic kidney failure patients with hyperphosphataemia: Randomised controlled trial. J. Clin. Nurs..

[31] Sulistyaningsih D.R., Nurachmah E., Yetti K., Hastono S.P. (2021). Nurses’ experience in improving adherence to fluid intake and diet in hemodialysis patients. Enferm. Clin..

[32] Wong F.K.Y., Chow S.K.Y., Chan T.M.F. (2010). Evaluation of a nurse-led disease management programme for chronic kidney disease: A randomized controlled trial. Int. J. Nurs. Stud..

[33] Bordin G., Zuccherato N., Toniato E. (2000). Nutritional education of CAPD patients and media influence. EDTNA-ERCA J..

[34] Cupisti A., Ferretti V., D’Alessandro C., Petrone I., Di Giorgio A., Meola M., Panichi V., Conti P., Lippi A., Caprioli R. (2012). Nutritional knowledge in hemodialysis patients and nurses: Focus on phosphorus. J. Ren. Nutr..

[35] Gazineo D., Andreoli D., Morales Palomares S., Palmisano A., Parozzi M., Mancin S., Salvini S., Godino L. (2025). Competenze dell’infermiere in ambito nefrologico in Italia: Un’indagine trasversale nazionale. G. Ital. Nefrol..

[36] Pafili Z., Maridaki M., Giannaki C.D., Karatzaferi C., Liakopoulos V., Eleftheriadis T., Stefanidis I., Sakkas G. (2019). Phosphorus nutritional knowledge among dialysis health care providers and patients: A multicenter observational study. Clin. Nutr. ESPEN.

[37] Sandlin K., Bennett P.N., Ockerby C., Corradini A.M. (2013). The impact of nurse-led education on haemodialysis patients’ phosphate binder medication adherence. J. Ren. Care.

[38] Xia Y.A., Healy A., Kruger R. (2016). Developing and validating a renal nutrition screening tool to effectively identify undernutrition risk among renal inpatients. J. Ren. Nutr..

[39] Ouirdani M., Boutib A., Fernane F.E., Hilali A., Saad E.M., Marfak A., Youlyouz-Marfak I. (2026). Bridging the nutritional care gap: Nurse-led education for potassium control in hemodialysis patients. Front. Nutr..

[40] Şanlıtürk D. (2026). Nurse-led animated video nutrition education and its effects on dietary knowledge and fluid–diet adherence in hemodialysis patients: A difference-in-differences analysis. Clin. Nurs. Res..

[41] Wang X.Z., Cheng G.M., Yu L.L., Zhao M. (2026). Competency of nurses in providing dietary guidance to patients with chronic kidney disease in Anhui Province: Influencing factors. J. Multidiscip. Healthc..

[42] Matteucci S., Ferrara G., Cangelosi G., Pozzuoli C., Morales Palomares S., Di Fronzo P., Grimaldi A., Durante A., Sguanci M., Mancin S. (2026). Nephrology Nurses’ Nutritional Competence in Chronic Kidney Disease Care: A Qualitative Study. Nurs. Rep..

[43] Shlipak M.G., Tummalapalli S.L., Boulware L.E., Grams M.E., Ix J.H., Jha V., Kengne A.P., Madero M., Mihaylova B., Tangri N. (2021). The case for early identification and intervention of chronic kidney disease: Conclusions from a Kidney Disease: Improving Global Outcomes (KDIGO) Controversies Conference. Kidney Int..

[44] Kang D., Youn S., Min J.W., Ko E.J. (2025). Nutritional status evaluation and intervention in chronic kidney disease patients: Practical approach. Nutrients.

[45] Lopez-Vargas P.A., Tong A., Howell M., Craig J.C. (2016). Educational interventions for patients with CKD: A systematic review. Am. J. Kidney Dis..

[46] Griva K., Nandakumar M., Ng J.A.H., Lam K.F.Y., McBain H., Newman S.P. (2018). Hemodialysis self-management intervention randomized trial (HED-SMART): A practical low-intensity intervention to improve adherence and clinical markers in patients receiving hemodialysis. Am. J. Kidney Dis..

[47] Ribeiro P.V.d.M., Hermsdorff H.H.M., Balbino K.P., Epifânio A.d.P.S., Jorge M.d.P., Moreira A.V.B. (2020). Effect of a nutritional intervention, based on transtheoretical model, on metabolic markers and food consumption of individuals undergoing hemodialysis. J. Ren. Nutr..

[48] Hunter E.G., Shukla A., Andrade J.M. (2023). Barriers to and strategies for dietary adherence: A qualitative study among hemodialysis/peritoneal dialysis patients and health care providers. J. Ren. Nutr..

[49] Llewellyn S. (2019). The chronic care model, kidney disease, and primary care: A scoping review. Nephrol. Nurs. J..

[50] Petrelli F., Cangelosi G., Nittari G., Pantanetti P., Debernardi G., Scuri S., Sagaro G.G., Nguyen C.T.T., Grappasonni I. (2021). Chronic Care Model in Italy: A narrative review of the literature. Prim. Health Care Res. Dev..

[51] Grudniewicz A., Gray C.S., Boeckxstaens P., De Maeseneer J., Mold J. (2023). Operationalizing the Chronic Care Model with Goal-Oriented Care. Patient.

[52] Jackson H. (2023). Enhancing nutrition screening in patients with kidney disease. Nurs. Stand..

[53] Hunn A. (2024). Embracing transformational leadership in nephrology nursing. Nephrol. Nurs. J..

[54] Paulus A.B., Kurosaka A. (2024). Transforming kidney health: The role of nursing leadership in value-based care. Nephrol. Nurs. J..

[55] Wouters O.J., O’Donoghue D.J., Ritchie J., Kanavos P.G., Narva A.S. (2015). Early chronic kidney disease: Diagnosis, management and models of care. Nat. Rev. Nephrol..

[56] Gapira B.E., Chironda G., Ndahayo D., Theos M.P.M., Tuyisenge M.J., Rajeswaran L. (2020). Knowledge related to chronic kidney disease (CKD) and perceptions on inpatient management practices among nurses at selected referral hospitals in Rwanda: A non-experimental descriptive correlational study. Int. J. Afr. Nurs. Sci..

[57] Tai Y.S., Foo Y.H., Ignacio J. (2024). Effectiveness of educational interventions for nurses caring for patients with chronic kidney disease in improving nurse outcomes: A systematic review. J. Clin. Nurs..

[58] Clark-Cutaia M.N., Sevick M.A., Thurheimer-Cacciotti J., Hoffman L.A., Snetselaar L., Burke L.E., Zickmund S.L. (2019). Perceived barriers to adherence to hemodialysis dietary recommendations. Clin. Nurs. Res..

[59] Yangöz Ş.T., Özer Z., Boz İ. (2021). Comparison of the effect of educational and self-management interventions on adherence to treatment in hemodialysis patients: A systematic review and meta-analysis of randomized controlled trials. Int. J. Clin. Pract..

[60] Lee H., Park H.H., Jo I.Y., Jhee J.H., Park J.T., Lee S.M. (2021). Effects of intensive individualized nutrition counseling on nutritional status and kidney function in patients with stage 3 and 4 chronic kidney disease. J. Ren. Nutr..

[61] Peng L., Gao Y., Lu R., Zhou R. (2020). Efficacy of Omaha system-based nursing management on nutritional status in patients undergoing peritoneal dialysis: A randomized controlled trial protocol. Medicine.

[62] Levin A., Ahmed S.B., Carrero J.J., Foster B., Francis A., Hall R.K., Herrington W.G., Hill G., Inker L.A., Kazancıoğlu R. (2024). Executive summary of the KDIGO 2024 Clinical Practice Guideline for the Evaluation and Management of Chronic Kidney Disease: Known knowns and known unknowns. Kidney Int..

[63] Bello A.K., Levin A., Tonelli M., Okpechi I.G., Feehally J., Harris D., Jindal K., Salako B.L., Rateb A., Osman M.A. (2017). Assessment of Global Kidney Health Care Status. JAMA.

[64] Molle J., Agnel J., Colson S., Chays-Amania A. (2025). Using implementation science to promote evidence-based nutritional care in healthcare settings: A mixed-methods systematic review. Int. J. Nurs. Stud. Adv..

[65] Choperena A., Rosa-Salas V., Esandi-Larramendi N., Diez-Del-Corral M.P., Jones D. (2025). Nursing educational framework: A new nurse-driven, conceptually guided approach. Int. J. Nurs. Knowl..

[66] Rawlinson C., Carron T., Cohidon C., Arditi C., Hong Q.N., Pluye P., Peytremann-Bridevaux I., Gilles I. (2021). An Overview of Reviews on Interprofessional Collaboration in Primary Care: Barriers and Facilitators. Int. J. Integr. Care.

[67] Robinson J., Jagiello K.P., McElroy L., LoVerde J., Jowell V., Good P., Brink S. (2024). Competency-Based Education in RN to BSN Programs. Nurse Educ..

[68] Palomares S.M., Ferrara G., Sguanci M., Gazineo D., Godino L., Palmisano A., Paderno A., Vrenna G., Faraglia E., Petrelli F. (2026). The Impact of Artificial Intelligence Technologies on Nutritional Care in Patients With Chronic Kidney Disease: A Systematic Review. J. Ren. Nutr..

[69] Hutter A., Brunner S., Gattinger H., Künzler-Heule P. (2026). ‘Malnutrition Receives High Priority’: Nurses’ Experiences in Providing Nutritional Support in Hospitalized Patients-A Qualitative Study. J. Clin. Nurs..

[70] Mancin S., Ferrara G., Lopane D., Cangelosi G., Petrelli F., Palomares S.M., Sguanci M. (2025). Artificial intelligence in the management of hospital malnutrition: A systematic review. Clin. Nutr. ESPEN.

[71] Abdelmohsen S.A., Al-Jabri M.M. (2025). Artificial Intelligence Applications in Healthcare: A Systematic Review of Their Impact on Nursing Practice and Patient Outcomes. J. Nurs. Scholarsh..

[72] Bekbolatova M., Mayer J., Ong C.W., Toma M. (2024). Transformative Potential of AI in Healthcare: Definitions, Applications, and Navigating the Ethical Landscape and Public Perspectives. Healthcare.

[73] Reddy S. (2024). Generative AI in healthcare: An implementation science informed translational path on application, integration and governance. Implement. Sci..

[74] Costa T., Borges-Tiago T., Martins F., Tiago F. (2025). System interoperability and data linkage in the era of health information management: A bibliometric analysis. Health Inf. Manag. J..

[75] D’Alleva A., Leigheb F., Rinaldi C., Di Stanislao F., Vanhaecht K., De Ridder D., Bruyneel L., Cangelosi G., Panella M. (2019). Achieving quadruple aim goals through clinical networks: A systematic review. J. Healthc. Qual. Res..

[76] Murazzano L., Landa P., Gartner J.B., Raki M.H., Côté A. (2026). Clinical pathways discovery for long-term and chronic patients: A process mining approach. Comput. Biol. Med..

[77] Sultan S., Morgan R.L., Murad M.H., Falck-Ytter Y., Dahm P., Schünemann H.J., Mustafa R.A. (2020). A Theoretical Framework and Competency-Based Approach to Training in Guideline Development. J. Gen. Intern. Med..

[78] Byrne A.L., Alrimawi I., Mirabal-Beltran R., Schultz T.R., Smith L., Roberts K.J. (2026). Global nurse navigation models in chronic disease care: A scoping review and conceptual framework development. Nurs. Outlook.

[79] Sheikh A., Anderson M., Albala S., Casadei B., Franklin B.D., Richards M., Taylor D., Tibble H., Mossialos E. (2021). Health information technology and digital innovation for national learning health and care systems. Lancet Digit. Health.

