# Supplementary material for: Mapping the Nutritional Roles, Competency Domains, and Practice Gaps of Nephrology Nurses in Chronic Kidney Disease: A Scoping Review
Source: Healthcare (Basel). 2026 Nov 6;14(21):3798. doi: 10.3390/healthcare14213798 (PMC13762861; doi:10.3390/healthcare14213798)
Supplement: Supplementary file 1 [file healthcare-14-03798-s001.zip › healthcare-4667219-supplementary.pdf]

Supplementary Files

S1. Preferred Reporting Items for Systematic Reviews and Meta-Analyses Extension for Scoping Reviews (PRISMA-ScR) Checklist

SECTION	ITEM	PRISMA-ScR CHECKLIST ITEM	REPORTED ON PAGE #
**TITLE**			
Title	1	Identify the report as a scoping review.	p. 1
**ABSTRACT**			
Structured summary	2	Provide a structured summary that includes (as applicable): background, objectives, eligibility criteria, sources of evidence, charting methods, results, and conclusions that relate to the review questions and objectives.	p. 2; Abstract
**INTRODUCTION**			
Rationale	3	Describe the rationale for the review in the context of what is already known. Explain why the review questions/objectives lend themselves to a scoping review approach.	pp. 2–3; Section 1 Introduction
Objectives	4	Provide an explicit statement of the questions and objectives being addressed with reference to their key elements (e.g., population or participants, concepts, and context) or other relevant key elements used to conceptualize the review questions and/or objectives.	p. 4; Section 1.1 Aims and Research Questions
**METHODS**			
Protocol and registration	5	Indicate whether a review protocol exists; state if and where it can be accessed (e.g., a Web address); and if available, provide registration information, including the registration number.	p. 4; Section 2.1 Protocol Registration and Reporting
Eligibility criteria	6	Specify characteristics of the sources of evidence used as eligibility criteria (e.g., years considered, language, and publication status), and provide a rationale.	p. 5; Section 2.3 Eligibility Criteria
Information sources*	7	Describe all information sources in the search (e.g., databases with dates of coverage and contact with authors to identify additional sources), as well as the date the most recent search was executed.	p. 5; Section 2.4 Search Strategy
Search	8	Present the full electronic search strategy for at least 1 database, including any limits used, such that it could be repeated.	p. 5; Section 2.4 Search Strategy; Supplementary File 2
Selection of sources of evidence†	9	State the process for selecting sources of evidence (i.e., screening and eligibility) included in the scoping review.	p. 5; Section 2.4 Search Strategy
Data charting process‡	10	Describe the methods of charting data from the included sources of evidence (e.g., calibrated forms or forms that have been tested by the team before their use, and whether data charting was done independently or in duplicate) and any processes for obtaining and confirming data from investigators.	p. 6; Section 2.5 Data Extraction Process
Data items	11	List and define all variables for which data were sought and any assumptions and simplifications made.	p. 6; Section 2.5 Data Extraction Process; Supplementary File 3
Critical appraisal of individual sources of evidence§	12	If done, provide a rationale for conducting a critical appraisal of included sources of evidence; describe the methods used and how this information was used in any data synthesis (if appropriate).	Not applicable. No formal critical appraisal or risk-of-bias assessment was undertaken, as the purpose of this scoping review was to map the extent, characteristics, and nature of the available evidence rather than to assess intervention effectiveness.
Synthesis of results	13	Describe the methods of handling and summarizing the data that were charted.	pp. 6–7; Section 2.6 Results Synthesis
**RESULTS**			
Selection of sources of evidence	14	Give numbers of sources of evidence screened, assessed for eligibility, and included in the review, with reasons for exclusions at each stage, ideally using a flow diagram.	p. 7; Section 3 Results; p. 8, Figure 1
Characteristics of sources of evidence	15	For each source of evidence, present characteristics for which data were charted and provide the citations.	pp. 8–14; Section 3.1 General Characteristics of the Studies Included; Table 1; Supplementary File 3
Critical appraisal within sources of evidence	16	If done, present data on critical appraisal of included sources of evidence (see item 12).	Not applicable. No formal critical appraisal or risk-of-bias assessment of the included sources was undertaken.
Results of individual	17	For each included source of evidence, present the relevant data that were	pp. 8–19; Sections 3.1–3.3; Tables 1–2;

SECTION	ITEM	PRISMA-ScR CHECKLIST ITEM	REPORTED ON PAGE #
sources of evidence		charted that relate to the review questions and objectives.	Supplementary File 3
Synthesis of results	18	Summarize and/or present the charting results as they relate to the review questions and objectives.	pp. 14–19; Sections 3.2–3.3; Table 2; Figure 2
**DISCUSSION**			
Summary of evidence	19	Summarize the main results (including an overview of concepts, themes, and types of evidence available), link to the review questions and objectives, and consider the relevance to key groups.	pp. 19–22; Section 4 Discussion
Limitations	20	Discuss the limitations of the scoping review process.	p. 22; Section 4.6 Strengths and Limitations
Conclusions	21	Provide a general interpretation of the results with respect to the review questions and objectives, as well as potential implications and/or next steps.	p. 23; Section 5 Conclusions
**FUNDING**			
Funding	22	Describe sources of funding for the included sources of evidence, as well as sources of funding for the scoping review. Describe the role of the funders of the scoping review.	Main manuscript, Funding section (p. 24): funding of the scoping review and funding information for all 20 included studies.

S2. Search Strategy

The following database-specific search strategies are reported based on the original search documentation available to the authors. Where archived platform search histories were available, the reported strategies were verified against those records. No additional filters or limits were applied.

The term “responsabil*” was used in the original database searches. We acknowledge that “responsib*” is the appropriate truncation for the intended terms “responsibility” and “responsible”. However, the original term has been retained to ensure accurate and transparent reporting of the search strategies actually performed.

ID	Cochrane Library<br><br>Search date: 23 May 2026; Platform: Cochrane Library; Limits: none.	Results
#1A	nurs* OR (registered NEXT nurse*) OR RN OR (staff NEXT nurs*) OR (clinical NEXT nurse NEXT specialist*) OR (nurse NEXT practitioner*) OR nursing OR (renal NEXT nurs*) in Title Abstract Keyword	
#2A	nephrolog* OR renal OR kidney* OR "chronic kidney disease" OR CKD OR "end-stage renal disease" OR ESRD OR ESKD OR (acute NEXT kidney NEXT injur*) OR AKI OR dialysis OR haemodialysis OR hemodialysis OR "peritoneal dialysis" OR (renal NEXT replacement NEXT therap*) OR RRT in Title Abstract Keyword	
#3A	competenc* OR competen* OR skill* OR abilit* OR knowledge* OR (knowledge NEXT level*) OR KAP OR expertise OR preparedness OR role* OR responsabil* OR "scope of practice" OR training OR education OR "continuing education" OR literacy OR "health literacy" in All Text	
#4A	"nutrition assessment" OR diet* OR malnutrition OR "diet therapy" OR "renal diet" OR "nutritional status" OR "protein-energy wasting" OR PEW OR MICS OR "malnutrition-inflammation complex syndrome" OR electrolyte* OR sodium OR potassium OR phosphate OR phosphorus OR calcium OR magnesium OR bicarbonate OR "acid-base" OR micronutrient* OR "vitamin D" OR cholecalciferol OR ergocalciferol OR calcitriol OR (vitamin NEXT B*) OR folate OR "folic acid" OR "vitamin B12" OR cobalamin OR "vitamin C" OR "ascorbic acid" OR "vitamin K" OR "vitamin A" OR retinol OR zinc OR selenium OR copper OR iodine OR iron OR ferritin OR transferrin OR "dietary protein" OR "protein intake" OR "energy intake" OR "caloric intake" OR albumin OR prealbumin OR "subjective global assessment" OR SGA OR "malnutrition-inflammation score" OR MIS in All Text	
#5A	**#1A AND #2A AND #3A AND #4A**	
#6A	**(nurs* OR (registered NEXT nurse*) OR RN OR (staff NEXT nurs*) OR (clinical NEXT nurse NEXT specialist*) OR (nurse NEXT practitioner*) OR nursing OR (renal NEXT nurs*)) in Title Abstract Keyword**<br>**AND**	**429**

	**(nephrolog* OR renal OR kidney* OR "chronic kidney disease" OR CKD OR "end-stage renal disease" OR ESRD OR ESKD OR (acute NEXT kidney NEXT injur*) OR AKI OR dialysis OR haemodialysis OR hemodialysis OR "peritoneal dialysis" OR (renal NEXT replacement NEXT therap*) OR RRT) in Title Abstract Keyword**<br>**AND**<br>**(competenc* OR competen* OR skill* OR abilit* OR knowledge* OR (knowledge NEXT level*) OR KAP OR expertise OR preparedness OR role* OR responsabil* OR "scope of practice" OR training OR education OR "continuing education" OR literacy OR "health literacy") in All Text**<br>**AND**<br>**("nutrition assessment" OR diet* OR malnutrition OR "diet therapy" OR "renal diet" OR "nutritional status" OR "protein-energy wasting" OR PEW OR MICS OR "malnutrition-inflammation complex syndrome" OR electrolyte* OR sodium OR potassium OR phosphate OR phosphorus OR calcium OR magnesium OR bicarbonate OR "acid-base" OR micronutrient* OR "vitamin D" OR cholecalciferol OR ergocalciferol OR calcitriol OR (vitamin NEXT B*) OR folate OR "folic acid" OR "vitamin B12" OR cobalamin OR "vitamin C" OR "ascorbic acid" OR "vitamin K" OR "vitamin A" OR retinol OR zinc OR selenium OR copper OR iodine OR iron OR ferritin OR transferrin OR "dietary protein" OR "protein intake" OR "energy intake" OR "caloric intake" OR albumin OR prealbumin OR "subjective global assessment" OR SGA OR "malnutrition-inflammation score" OR MIS) in All Text**	
	**Pubmed**<br><br>**Search date: 23 May 2026; Platform: PubMed; Limits: none.**	**Results**
**#1A**	("Nurses"[Mesh] OR "Nursing"[Mesh] OR "nurse*"[tiab] OR nursing[tiab] OR "registered nurse*"[tiab] OR RN [tiab] OR "staff nurs*"[tiab] OR "clinical nurse specialist*"[tiab] OR "nurse practitioner*"[tiab] OR nurs*[tiab] OR "renal nurs*"[tiab])	
**#2A**	("Nephrology"[Mesh] OR "Kidney Diseases"[Mesh] OR "Renal Dialysis"[Mesh] OR nephrolog*[tiab] OR renal[tiab] OR kidney*[tiab] OR "chronic kidney disease"[tiab] OR CKD [tiab] OR "end-stage renal disease"[tiab] OR ESRD [tiab] OR ESKD [tiab] OR "acute kidney injur*"[tiab] OR AKI [tiab] OR dialysis[tiab] OR haemodialysis[tiab] OR hemodialysis[tiab] OR "peritoneal dialysis"[tiab] OR "renal replacement therap*"[tiab] OR RRT [tiab])	
**#3A**	(competenc* OR competen* OR skill* OR abilit* OR knowledge* OR "knowledge level*" OR KAP OR expertise OR preparedness OR role* OR responsabil* OR "scope of practice" OR training OR education OR "continuing education" OR literacy OR "health literacy")	
**#4A**	("Nutrition Assessment"[Mesh] OR Diet[Mesh] OR "Malnutrition"[Mesh] OR "Diet Therapy"[Mesh] OR Diet, Renal OR "Micronutrients"[Mesh] OR "Electrolytes"[Mesh] OR "Vitamins"[Mesh] OR "Iron"[Mesh] OR nutrition* OR diet* OR "medical nutrition therap*" OR "renal diet" OR "nutritional status" OR malnutrition OR "protein-energy wasting" OR PEW OR MICS OR "malnutrition-inflammation complex syndrome" OR electrolyte* OR sodium OR potassium OR phosphate OR phosphorus OR calcium OR magnesium OR bicarbonate OR "acid-base" OR micronutrient* OR "vitamin D" OR cholecalciferol OR ergocalciferol OR calcitriol OR "vitamin B*" OR folate OR "folic acid" OR "vitamin B12" OR cobalamin OR "vitamin C" OR "ascorbic acid" OR "vitamin K" OR "vitamin A" OR retinol OR zinc OR selenium OR copper OR iodine OR iron OR ferritin OR transferrin OR "dietary protein" OR "protein intake" OR "energy intake" OR "caloric intake" OR albumin OR prealbumin OR "Subjective Global Assessment" OR SGA OR "malnutrition-inflammation score" OR MIS)	
**#5A**	**#1A AND #2A AND #3A AND #4A**	
**#6A**	**Search: (((("Nurses"[Mesh] OR "Nursing"[Mesh] OR "nurse*"[tiab] OR nursing[tiab] OR "registered nurse*"[tiab] OR RN[tiab] OR "staff nurs*"[tiab] OR "clinical nurse specialist*"[tiab] OR "nurse practitioner*"[tiab] OR nurs*[tiab] OR "renal nurs*"[tiab])) AND ("Nephrology"[Mesh] OR "Kidney Diseases"[Mesh] OR "Renal Dialysis"[Mesh] OR nephrolog*[tiab] OR renal[tiab] OR kidney*[tiab] OR "chronic kidney disease"[tiab] OR CKD[tiab] OR "end-stage renal disease"[tiab] OR ESRD[tiab] OR ESKD[tiab] OR "acute kidney injur*"[tiab] OR AKI[tiab] OR dialysis[tiab] OR haemodialysis[tiab] OR hemodialysis[tiab] OR "peritoneal dialysis"[tiab] OR "renal replacement therap*"[tiab] OR RRT[tiab])) AND ((competenc* OR competen* OR skill* OR abilit* OR knowledge* OR "knowledge level*" OR KAP OR expertise OR preparedness OR role* OR responsabil* OR "scope of practice" OR training OR education OR "continuing education" OR literacy OR "health literacy"))) AND (("Nutrition Assessment"[Mesh] OR Diet[Mesh] OR "Malnutrition"[Mesh] OR "Diet Therapy"[Mesh] OR Diet, Renal OR "Micronutrients"[Mesh] OR "Electrolytes"[Mesh] OR "Vitamins"[Mesh] OR "Iron"[Mesh] OR nutrition* OR diet* OR "medical nutrition therap*" OR "renal diet" OR "nutritional status" OR malnutrition OR "protein-energy wasting" OR PEW OR MICS OR "malnutrition-inflammation complex syndrome" OR electrolyte* OR sodium OR potassium OR phosphate OR phosphorus OR calcium OR magnesium OR bicarbonate OR "acid-base" OR micronutrient* OR "vitamin D" OR cholecalciferol OR ergocalciferol OR**	**1,062**

	**calcitriol OR "vitamin B*" OR folate OR "folic acid" OR "vitamin B12" OR cobalamin OR "vitamin C" OR "ascorbic acid" OR "vitamin K" OR "vitamin A" OR retinol OR zinc OR selenium OR copper OR iodine OR iron OR ferritin OR transferrin OR "dietary protein" OR "protein intake" OR "energy intake" OR "caloric intake" OR albumin OR prealbumin OR "Subjective Global Assessment" OR SGA OR "malnutrition-inflammation score" OR MIS))**	
**ID**	**Cumulative Index Of Nursing and Allied Health Literature (CINAHL)**<br><br>**Search date: 23 May 2026; Platform: EBSCOhost; Limits: none.**	**Results**
**#1A**	XB (MH "Nurses" OR MH "Nursing" OR nurse* OR nursing OR "registered nurse*" OR RN OR "staff nurs*" OR "clinical nurse specialist*" OR "nurse practitioner*" OR nurs* OR "renal nurs*")	
**#2A**	XB (MH "Nephrology" OR MH "Kidney Diseases" OR MH "Renal Dialysis" OR nephrolog* OR renal OR kidney* OR "chronic kidney disease" OR CKD OR "end-stage renal disease" OR ESRD OR ESKD OR "acute kidney injur*" OR AKI OR dialysis OR haemodialysis OR hemodialysis OR "peritoneal dialysis" OR "renal replacement therap*" OR RRT)	
**#3A**	(competenc* OR competen* OR skill* OR abilit* OR knowledge* OR "knowledge level*" OR KAP OR expertise OR preparedness OR role* OR responsabil* OR "scope of practice" OR training OR education OR "continuing education" OR literacy OR "health literacy")	
**#4A**	(MH "Nutrition Assessment" OR MH "Malnutrition" OR MH "Diet Therapy" OR MH "Diet, Renal" OR MH "Micronutrients" OR MH "Electrolytes" OR MH "Vitamins" OR MH "Iron" OR nutrition* OR diet* OR "medical nutrition therap*" OR "renal diet" OR "nutritional status" OR malnutrition OR "protein-energy wasting" OR PEW OR MICS OR "malnutrition-inflammation complex syndrome" OR electrolyte* OR sodium OR potassium OR phosphate OR phosphorus OR calcium OR magnesium OR bicarbonate OR "acid-base" OR micronutrient* OR "vitamin D" OR cholecalciferol OR ergocalciferol OR calcitriol OR "vitamin B*" OR folate OR "folic acid" OR "vitamin B12" OR cobalamin OR "vitamin C" OR "ascorbic acid" OR "vitamin K" OR "vitamin A" OR retinol OR zinc OR selenium OR copper OR iodine OR iron OR ferritin OR transferrin OR "dietary protein" OR "protein intake" OR "energy intake" OR "caloric intake" OR albumin OR prealbumin OR "Subjective Global Assessment" OR SGA OR "malnutrition-inflammation score" OR MIS)	
**#5A**	**#1A AND #2A AND #3A AND #4A**	
**#6A**	**XB ((MH "Nurses" OR MH "Nursing" OR nurse* OR nursing OR "registered nurse*" OR RN OR "staff nurs*" OR "clinical nurse specialist*" OR "nurse practitioner*" OR nurs* OR "renal nurs*")) AND XB ((MH "Nephrology" OR MH "Kidney Diseases" OR MH "Renal Dialysis" OR nephrolog* OR renal OR kidney* OR "chronic kidney disease" OR CKD OR "end-stage renal disease" OR ESRD OR ESKD OR "acute kidney injur*" OR AKI OR dialysis OR haemodialysis OR hemodialysis OR "peritoneal dialysis" OR "renal replacement therap*" OR RRT)) AND ((competenc* OR competen* OR skill* OR abilit* OR knowledge* OR "knowledge level*" OR KAP OR expertise OR preparedness OR role* OR responsabil* OR "scope of practice" OR training OR education OR "continuing education" OR literacy OR "health literacy")) AND ((MH "Nutrition Assessment" OR MH "Malnutrition" OR MH "Diet Therapy" OR MH "Diet, Renal" OR MH "Micronutrients" OR MH "Electrolytes" OR MH "Vitamins" OR MH "Iron" OR nutrition* OR diet* OR "medical nutrition therap*" OR "renal diet" OR "nutritional status" OR malnutrition OR "protein-energy wasting" OR PEW OR MICS OR "malnutrition-inflammation complex syndrome" OR electrolyte* OR sodium OR potassium OR phosphate OR phosphorus OR calcium OR magnesium OR bicarbonate OR "acid-base" OR micronutrient* OR "vitamin D" OR cholecalciferol OR ergocalciferol OR calcitriol OR "vitamin B*" OR folate OR "folic acid" OR "vitamin B12" OR cobalamin OR "vitamin C" OR "ascorbic acid" OR "vitamin K" OR "vitamin A" OR retinol OR zinc OR selenium OR copper OR iodine OR iron OR ferritin OR transferrin OR "dietary protein" OR "protein intake" OR "energy intake" OR "caloric intake" OR albumin OR prealbumin OR "Subjective Global Assessment" OR SGA OR "malnutrition-inflammation score" OR MIS))**	**679**
**ID**	**EMBASE**<br><br>**Search date: 23 May 2026; Platform: Embase.com (Elsevier); Limits: none.**	**Results**
**#1A**	(nurse*:ti OR nurs*:ti OR nursing:ti OR "registered nurse*":ti OR RN:ti OR "staff nurs*":ti OR "clinical nurse specialist*":ti OR "nurse practitioner*":ti OR "renal nurs*":ti)	
**#2A**	(nephrolog*:ti OR renal:ti OR kidney*:ti OR "chronic kidney disease":ti OR CKD:ti OR "end stage renal disease":ti OR ESRD:ti OR ESKD:ti	

	OR "acute kidney injur*":ti OR AKI:ti OR dialysis:ti OR haemodialysis:ti OR hemodialysis:ti OR "peritoneal dialysis":ti OR "renal replacement therap*":ti OR RRT:ti)	
**#3A**	(competenc*:ti,ab,kw,de OR competen*:ti,ab,kw,de OR skill*:ti,ab,kw,de OR abilit*:ti,ab,kw,de OR knowledge*:ti,ab,kw,de OR "knowledge level*":ti,ab,kw,de OR KAP:ti,ab,kw,de OR expertise:ti,ab,kw,de OR preparedness:ti,ab,kw,de OR role*:ti,ab,kw,de OR responsabil*:ti,ab,kw,de OR "scope of practice":ti,ab,kw,de OR training:ti,ab,kw,de OR education:ti,ab,kw,de OR "continuing education":ti,ab,kw,de OR literacy:ti,ab,kw,de OR "health literacy":ti,ab,kw,de)	
**#4A**	(nutrition*:ti,ab,kw,de OR diet*:ti,ab,kw,de OR "medical nutrition therap*":ti,ab,kw,de OR "renal diet":ti,ab,kw,de OR "nutritional status":ti,ab,kw,de OR malnutrition:ti,ab,kw,de OR "protein energy wasting":ti,ab,kw,de OR PEW:ti,ab,kw,de OR MICS:ti,ab,kw,de OR "malnutrition inflammation complex syndrome":ti,ab,kw,de OR electrolyte*:ti,ab,kw,de OR sodium:ti,ab,kw,de OR potassium:ti,ab,kw,de OR phosphate:ti,ab,kw,de OR phosphorus:ti,ab,kw,de OR calcium:ti,ab,kw,de OR magnesium:ti,ab,kw,de OR bicarbonate:ti,ab,kw,de OR "acid base":ti,ab,kw,de OR micronutrient*:ti,ab,kw,de OR "vitamin D":ti,ab,kw,de OR cholecalciferol:ti,ab,kw,de OR ergocalciferol:ti,ab,kw,de OR calcitriol:ti,ab,kw,de OR "vitamin B*":ti,ab,kw,de OR folate:ti,ab,kw,de OR "folic acid":ti,ab,kw,de OR "vitamin B12":ti,ab,kw,de OR cobalamin:ti,ab,kw,de OR "vitamin C":ti,ab,kw,de OR "ascorbic acid":ti,ab,kw,de OR "vitamin K":ti,ab,kw,de OR "vitamin A":ti,ab,kw,de OR retinol:ti,ab,kw,de OR zinc:ti,ab,kw,de OR selenium:ti,ab,kw,de OR copper:ti,ab,kw,de OR iodine:ti,ab,kw,de OR iron:ti,ab,kw,de OR ferritin:ti,ab,kw,de OR transferrin:ti,ab,kw,de OR "dietary protein":ti,ab,kw,de OR "protein intake":ti,ab,kw,de OR "energy intake":ti,ab,kw,de OR "caloric intake":ti,ab,kw,de OR albumin:ti,ab,kw,de OR prealbumin:ti,ab,kw,de OR "subjective global assessment":ti,ab,kw,de OR SGA:ti,ab,kw,de OR "malnutrition inflammation score":ti,ab,kw,de OR MIS:ti,ab,kw,de)	
**#5A**	**#1A AND #2A AND #3A AND #4A**	
**#6A**	**(nurse*:ti OR nurs*:ti OR nursing:ti OR "registered nurse*":ti OR RN:ti OR "staff nurs*":ti OR "clinical nurse specialist*":ti OR "nurse practitioner*":ti OR "renal nurs*":ti) AND (nephrolog*:ti OR renal:ti OR kidney*:ti OR "chronic kidney disease":ti OR CKD:ti OR "end stage renal disease":ti OR ESRD:ti OR ESKD:ti OR "acute kidney injur*":ti OR AKI:ti OR dialysis:ti OR haemodialysis:ti OR hemodialysis:ti OR "peritoneal dialysis":ti OR "renal replacement therap*":ti OR RRT:ti) AND (competenc*:ti,ab,kw,de OR competen*:ti,ab,kw,de OR skill*:ti,ab,kw,de OR abilit*:ti,ab,kw,de OR knowledge*:ti,ab,kw,de OR "knowledge level*":ti,ab,kw,de OR KAP:ti,ab,kw,de OR expertise:ti,ab,kw,de OR preparedness:ti,ab,kw,de OR role*:ti,ab,kw,de OR responsabil*:ti,ab,kw,de OR "scope of practice":ti,ab,kw,de OR training:ti,ab,kw,de OR education:ti,ab,kw,de OR "continuing education":ti,ab,kw,de OR literacy:ti,ab,kw,de OR "health literacy":ti,ab,kw,de) AND (nutrition*:ti,ab,kw,de OR diet*:ti,ab,kw,de OR "medical nutrition therap*":ti,ab,kw,de OR "renal diet":ti,ab,kw,de OR "nutritional status":ti,ab,kw,de OR malnutrition:ti,ab,kw,de OR "protein energy wasting":ti,ab,kw,de OR PEW:ti,ab,kw,de OR MICS:ti,ab,kw,de OR "malnutrition inflammation complex syndrome":ti,ab,kw,de OR electrolyte*:ti,ab,kw,de OR sodium:ti,ab,kw,de OR potassium:ti,ab,kw,de OR phosphate:ti,ab,kw,de OR phosphorus:ti,ab,kw,de OR calcium:ti,ab,kw,de OR magnesium:ti,ab,kw,de OR bicarbonate:ti,ab,kw,de OR "acid base":ti,ab,kw,de OR micronutrient*:ti,ab,kw,de OR "vitamin D":ti,ab,kw,de OR cholecalciferol:ti,ab,kw,de OR ergocalciferol:ti,ab,kw,de OR calcitriol:ti,ab,kw,de OR "vitamin B*":ti,ab,kw,de OR folate:ti,ab,kw,de OR "folic acid":ti,ab,kw,de OR "vitamin B12":ti,ab,kw,de OR cobalamin:ti,ab,kw,de OR "vitamin C":ti,ab,kw,de OR "ascorbic acid":ti,ab,kw,de OR "vitamin K":ti,ab,kw,de OR "vitamin A":ti,ab,kw,de OR retinol:ti,ab,kw,de OR zinc:ti,ab,kw,de OR selenium:ti,ab,kw,de OR copper:ti,ab,kw,de OR iodine:ti,ab,kw,de OR iron:ti,ab,kw,de OR ferritin:ti,ab,kw,de OR transferrin:ti,ab,kw,de OR "dietary protein":ti,ab,kw,de OR "protein intake":ti,ab,kw,de OR "energy intake":ti,ab,kw,de OR "caloric intake":ti,ab,kw,de OR albumin:ti,ab,kw,de OR prealbumin:ti,ab,kw,de OR "subjective global assessment":ti,ab,kw,de OR SGA:ti,ab,kw,de OR "malnutrition inflammation score":ti,ab,kw,de OR MIS:ti,ab,kw,de)**	**227**
**ID**	**SCOPUS**<br><br>**Search date: 23 May 2026; Platform: Scopus; Limits: none.**	**Results**
#1A	TITLE-ABS-KEY (nurse* OR nursing OR nurs* OR RN OR "registered nurse" OR "registered nurses" OR "staff nurse" OR "staff nurses" OR "clinical nurse specialist" OR "clinical nurse specialists" OR "nurse practitioner" OR "nurse practitioners" OR "renal nurse" OR "renal nurses" OR "renal nursing")	
#2A	TITLE-ABS-KEY (nephrolog* OR renal OR kidney* OR "chronic kidney disease" OR CKD OR "end-stage renal disease" OR ESRD OR ESKD OR "acute kidney injury" OR "acute kidney injuries" OR AKI OR dialysis OR haemodialysis OR hemodialysis OR "peritoneal dialysis" OR	

	"renal replacement therapy" OR "renal replacement therapies" OR RRT)	
#3A	TITLE-ABS-KEY (competenc* OR competen* OR skill* OR abilit* OR knowledge* OR "knowledge level" OR "knowledge levels" OR KAP OR expertise OR preparedness OR role* OR responsabil* OR "scope of practice" OR training OR education OR "continuing education" OR literacy OR "health literacy")	
**#4A**	TITLE-ABS-KEY ("nutrition assessment" OR diet* OR malnutrition OR "diet therapy" OR "renal diet" OR "nutritional status" OR "protein-energy wasting" OR PEW OR MICS OR "malnutrition-inflammation complex syndrome" OR electrolyte* OR sodium OR potassium OR phosphate OR phosphorus OR calcium OR magnesium OR bicarbonate OR "acid-base" OR micronutrient* OR "vitamin D" OR cholecalciferol OR ergocalciferol OR calcitriol OR "vitamin B" OR "vitamin B1" OR "vitamin B6" OR "vitamin B12" OR folate OR "folic acid" OR cobalamin OR "vitamin C" OR "ascorbic acid" OR "vitamin K" OR "vitamin A" OR retinol OR zinc OR selenium OR copper OR iodine OR iron OR ferritin OR transferrin OR "dietary protein" OR "protein intake" OR "energy intake" OR "caloric intake" OR albumin OR prealbumin OR "subjective global assessment" OR SGA OR "malnutrition-inflammation score" OR MIS)	
**#5A**	**#1A AND #2A AND #3A AND #4A**	
**#6B**	(TITLE-ABS-KEY ((nurse* OR nursing OR nurs* OR RN OR "registered nurse" OR "registered nurses" OR "staff nurse" OR "staff nurses" OR "clinical nurse specialist" OR "clinical nurse specialists" OR "nurse practitioner" OR "nurse practitioners" OR "renal nurse" OR "renal nurses" OR "renal nursing")) AND TITLE-ABS-KEY ((nephrolog* OR renal OR kidney* OR "chronic kidney disease" OR CKD OR "end-stage renal disease" OR ESRD OR ESKD OR "acute kidney injury" OR "acute kidney injuries" OR AKI OR dialysis OR haemodialysis OR hemodialysis OR "peritoneal dialysis" OR "renal replacement therapy" OR "renal replacement therapies" OR RRT)) AND TITLE-ABS-KEY ((competenc* OR competen* OR skill* OR abilit* OR knowledge* OR "knowledge level" OR "knowledge levels" OR KAP OR expertise OR preparedness OR role* OR responsabil* OR "scope of practice" OR training OR education OR "continuing education" OR literacy OR "health literacy")) AND TITLE-ABS-KEY (("nutrition assessment" OR diet* OR malnutrition OR "diet therapy" OR "renal diet" OR "nutritional status" OR "protein-energy wasting" OR PEW OR MICS OR "malnutrition-inflammation complex syndrome" OR electrolyte* OR sodium OR potassium OR phosphate OR phosphorus OR calcium OR magnesium OR bicarbonate OR "acid-base" OR micronutrient* OR "vitamin D" OR cholecalciferol OR ergocalciferol OR calcitriol OR "vitamin B" OR "vitamin B1" OR "vitamin B6" OR "vitamin B12" OR folate OR "folic acid" OR cobalamin OR "vitamin C" OR "ascorbic acid" OR "vitamin K" OR "vitamin A" OR retinol OR zinc OR selenium OR copper OR iodine OR iron OR ferritin OR transferrin OR "dietary protein" OR "protein intake" OR "energy intake" OR "caloric intake" OR albumin OR prealbumin OR "subjective global assessment" OR SGA OR "malnutrition-inflammation score" OR MIS))	**1,702**
ID	**Web of Science**<br><br>**Search date: 23 May 2026; Platform: Web of Science (Clarivate); Limits: none**	Results
**#1A**	Title=(nurse* OR nurs* OR nursing OR "registered nurse*" OR RN OR "staff nurs*" OR "clinical nurse specialist*" OR "nurse practitioner*" OR "renal nurs*")	
**#2A**	Title=(nephrolog* OR renal OR kidney* OR "chronic kidney disease" OR CKD OR "end stage renal disease" OR ESRD OR ESKD OR "acute kidney injur*" OR AKI OR dialysis OR haemodialysis OR hemodialysis OR "peritoneal dialysis" OR "renal replacement therap*" OR RRT)	
**#3A**	Topic=(competenc* OR competen* OR skill* OR abilit* OR knowledge* OR "knowledge level*" OR KAP OR expertise OR preparedness OR role* OR responsabil* OR "scope of practice" OR training OR education OR "continuing education" OR literacy OR "health literacy")	
**#4A**	Topic=(nutrition* OR diet* OR "medical nutrition therap*" OR "renal diet" OR "nutritional status" OR malnutrition OR "protein energy wasting" OR PEW OR MICS OR "malnutrition inflammation complex syndrome" OR electrolyte* OR sodium OR potassium OR phosphate OR phosphorus OR calcium OR magnesium OR bicarbonate OR "acid base" OR micronutrient* OR "vitamin D" OR cholecalciferol OR ergocalciferol OR calcitriol OR "vitamin B*" OR folate OR "folic acid" OR "vitamin B12" OR cobalamin OR "vitamin C" OR "ascorbic acid" OR "vitamin K" OR "vitamin A" OR retinol OR zinc OR selenium OR copper OR iodine OR iron OR ferritin OR transferrin OR "dietary protein" OR "protein intake" OR "energy intake" OR "caloric intake" OR albumin OR prealbumin OR "subjective global assessment" OR SGA OR "malnutrition inflammation score" OR MIS)	
**#5A**	**#1A AND #2A AND #3A AND #4A**	

**#6B**	Title=(nurse* OR nurs* OR nursing OR "registered nurse*" OR RN OR "staff nurs*" OR "clinical nurse specialist*" OR "nurse practitioner*" OR "renal nurs*") AND Title=(nephrolog* OR renal OR kidney* OR "chronic kidney disease" OR CKD OR "end stage renal disease" OR ESRD OR ESKD OR "acute kidney injur*" OR AKI OR dialysis OR haemodialysis OR hemodialysis OR "peritoneal dialysis" OR "renal replacement therap*" OR RRT) AND Topic=(competenc* OR competen* OR skill* OR abilit* OR knowledge* OR "knowledge level*" OR KAP OR expertise OR preparedness OR role* OR responsabil* OR "scope of practice" OR training OR education OR "continuing education" OR literacy OR "health literacy") AND Topic=(nutrition* OR diet* OR "medical nutrition therap*" OR "renal diet" OR "nutritional status" OR malnutrition OR "protein energy wasting" OR PEW OR MICS OR "malnutrition inflammation complex syndrome" OR electrolyte* OR sodium OR potassium OR phosphate OR phosphorus OR calcium OR magnesium OR bicarbonate OR "acid base" OR micronutrient* OR "vitamin D" OR cholecalciferol OR ergocalciferol OR calcitriol OR "vitamin B*" OR folate OR "folic acid" OR "vitamin B12" OR cobalamin OR "vitamin C" OR "ascorbic acid" OR "vitamin K" OR "vitamin A" OR retinol OR zinc OR selenium OR copper OR iodine OR iron OR ferritin OR transferrin OR "dietary protein" OR "protein intake" OR "energy intake" OR "caloric intake" OR albumin OR prealbumin OR "subjective global assessment" OR SGA OR "malnutrition inflammation score" OR MIS)	**175**
**1#**	**Google Scholar**<br><br>**Search date: 23 May 2026; Results screened: 425; Ordering: default relevance; Limits: none.**<br><br>**("nurses" OR "nursing staff") AND (nephrology OR dialysis) AND ("nutrition knowledge" OR "nutrition competence" OR "nutrition training") AND ("chronic kidney disease" OR CKD OR ESRD)**	**425**
		**Total**<br><br>**4,699**

S3. Complete data extraction matrix of the included studies

Study	Country	Design	Setting	Participants	N	CKD stage / modality	Aim	Nursing role / intervention	Competency construct	Instrument	Outcomes	Main findings	Practice / educational gaps	Domain
**Ferrara et al., 2026 [18]**	Italy	Methodological psychometric validation	Nephrology and dialysis settings nationwide	Nephrology nurses	405	CKD/ESRD; nephrology, HD, PD, transplant, and nephrology palliative care settings	Develop and validate NECN-ESRD	No clinical intervention; competency assessment study	Self-reported nutritional nursing competence	NECN-ESRD	Validity, reliability, competency dimensions	Four-factor structure; strong psychometric properties	Lack of an ESRD-specific validated competency assessment tool	Clinical and advanced
**Ouirdani et al., 2026 [39]**	Morocco	Quasi-experimental pre–post pilot	Three public HD centers without dietitians	Chronic HD patients	30	ESRD; maintenance HD	Assess feasibility of nurse-led education for potassium control	Nurse-led nutrition and lifestyle education	Not directly assessed; educational nursing role	Serum K^+; IDWG; EQ-5D-5L	Serum K^+, QoL, IDWG	Improved potassium control; no significant QoL or IDWG changes	Limited access to dietitians and individualized nutritional counseling	Educational and communicative
**Şanlıtürk et al., 2026 [40]**	Turkey	Nonrandomized pre–post controlled study	Two public HD units	Adult HD patients	107	CKD; maintenance HD	Assess nurse-led animated nutrition education	Nurse-led animated video nutrition education	Not directly assessed; educational nursing role	Nutritional knowledge survey; DDFQ; clinical measures	Nutritional knowledge, diet–fluid adherence, clinical parameters	Improved nutritional knowledge and diet–fluid adherence	Need for more structured and accessible nutrition education	Educational and communicative
**Wang et al., 2026 [41]**	China	Cross-sectional	36 secondary/tertiary	Frontline clinical nurses	393 Clinical nurses (139 nephrology, 104 blood	CKD care; nephrology, HD, endocrinol	Assess CKD dietary guidance competency	Dietary guidance competency	Self-reported dietary guidance	CKD Dietary Guidance Competency Assessment	Competency scores and associated	Moderate competency in the overall	Limited formal nutrition training	Clinical and advanced; Guidance

			hospitals		purification, 59 endocrinology, 91 urological surgery)	ogy, and urology	and influencing factors	assessment	competency	Form	factors	mixed nursing sample; practice scored lowest; competency associated with training and organizational factors	and need to strengthen practical dietary guidance	and counseling
**Baraz et al., 2010 [24]**	Iran	RCT	Three general hospital HD units	HD patients	63	ESRD; HD	Assess educational interventions on dietary and fluid compliance	Oral/video dietary and fluid education	Not directly assessed; educational nursing role	Biochemical measures; IDWG; compliance assessment	Dietary/fluid compliance, biochemical parameters, IDWG	Oral and video education improved dietary/fluid compliance; no difference between approaches	Need to reinforce dietary and fluid education, particularly sodium restriction	Educational and communicative
**Bennett et al., 2013 [14]**	Australia	RCT	Four satellite HD centers	HD patients	81	CKD stage 5; HD	Assess whether nurse-led nutritional screening increases dietetic referrals and affects outcomes	Monthly nurse-completed nutritional screening and referral	Not directly assessed; nutritional screening/referral role	Validated 4-item nutrition screening tool	Dietetic referrals, biochemical indices, BP, mortality, QoL	Screening tripled dietetic referrals; no meaningful effect on mortality or QoL	Limited dietetic support; need for systematic nutritional screening and referral	Clinical and advanced
**Bordin et al., 2000 [33]**	Italy	Quasi-experimental pre-post	Peritoneal Dialysis Center	CAPD patients and family members	22 patients + 17 family members	ESRD; CAPD	Assess nutritional knowledge and	Multidisciplinary group nutritional education	Not directly assessed; educational nursing role	Pre/post nutritional questionnaire; media	Nutritional knowledge; influence of media	Group meetings improved nutritional	Need for continuing nutrition education	Collaborative and professional

		study					usefulness of group education	involving nurses		questionnaire		knowledge and highlighted media influence	beyond initial PD training; misinformation from media	
**Cupisti et al., 2012 [34]**	Italy	Cross-sectional cohort study	Five HD centers in Tuscany	HD patients, dialysis nurses, and non-healthcare controls	191 patients; 105 nurses; 86 controls	ESRD; maintenance HD	Assess phosphorus and other nutrient knowledge in patients and nurses	Nutritional knowledge assessment	Nutritional knowledge	25-item CKDKAT-N	Phosphorus and overall nutritional knowledge scores	Nurses scored higher than patients but showed important phosphorus knowledge gaps	Insufficient phosphorus knowledge; need for nutrition training	General or Basic Nutritional
**Emran & Zolkefli, 2021 [28]**	Brunei Darussalam	Qualitative descriptive study	One renal/dialysis center	Nephrology nurses	12	Dialysis patients cared for by participating nurses	Explore nurses' views on providing dietary recommendations	Dietary guidance, adherence support, and negotiation	Perceived dietary guidance practice	Semi-structured interviews and focus group	Themes related to dietary guidance practices and experiences	Three themes: information approaches, adherence promotion, and negotiation	Current dietary guidance methods perceived as improvable; lack of standardized advice	Guidance and counseling
**Fernandes et al., 2021 [25]**	India	Uncontrolled before–after study	Hospital setting; participating nurses◎ departments not reported	Hospital staff nurses; nephrology affiliation not specified	40	CKD care	Evaluate a teaching program on nurses' nutritional knowledge	60-min planned teaching program for nurses	Assessed CKD-specific nutritional knowledge; indirect evidence for nephrology nursing	Structured knowledge questionnaire	Nurses' nutritional knowledge scores	Knowledge significantly improved after the teaching program	Baseline knowledge was mainly average/poor; need for structured education	Educational and communicative
**Gaber, 2022 [26]**	Iraq	Descriptive cross-sectional study	Three hospital HD centers	Nurses working in HD centers	80	ESRD; HD	Assess nurses' knowledge of biochemical parameters	Nutritional status assessment	Nutritional assessment knowledge	Researcher-developed knowledge questionnaire	Knowledge of biochemical and dietary assessment	Most nurses showed poor knowledge;	Poor nutritional assessment knowledge; need for	General or Basic Nutritional

							and dietary assessment					differences by age and experience	targeted education	
**Gazineo et al., 2025 [35]**	Italy	Cross-sectional study	Nephrology settings nationwide	Nephrology nurses	296	Nephrology care; predominantly HD	Explore basic and advanced nephrology nursing competencies	Competency assessment	Self-reported nephrology competencies, including nutrition	Ad hoc validated questionnaire	Theoretical knowledge, practical competencies, professional development	Nutrition competence was medium/low in 35%; communication and decision-making scored higher	Limited post-basic training; need for further nutrition and specialist education	Clinical and advanced; Collaborative and professional
**Irzaij & Atiyah, 2021 [27]**	Iraq	Cross-sectional study	Dialysis units/wards of Al-Hussain Teaching Hospital	Dialysis nurses	100	ESRD; dialysis	Assess nurses' knowledge regarding nutritional status in ESRD	Nutritional status knowledge assessment	Nutritional knowledge	Interview questionnaire	Nurses' nutritional knowledge and associated characteristics	Most nurses had moderate knowledge; higher education was associated with better knowledge	Need to strengthen nurses' nutritional knowledge in ESRD care	General or Basic Nutritional
**Pafili et al., 2019 [36]**	Greece	Cross-sectional study	Three HD units	HD patients, renal nurses, and nephrologists	68 patients; 19 nurses; 11 nephrologists	ESRD; HD	Assess phosphorus nutritional knowledge among patients and dialysis professionals	Nutritional knowledge assessment	Phosphorus and renal nutrition knowledge	25-item CKDKATeN	Total and phosphorus-specific knowledge scores	Nurses had low phosphorus knowledge, similar to patients; nephrologists scored higher	Major phosphorus knowledge gaps; need for continuing nutrition education	General or Basic Nutritional
**Rasheed et al., 2018 [29]**	Iraq	Cross-sectional study	Two dialysis centers in Erbil	HD nurses	51	Renal failure; HD	Assess nurses' knowledge of nutritional management in renal failure	Nutritional management knowledge assessment	Nutritional knowledge	14-item self-reported knowledge questionnaire	Nutritional knowledge scores and associated characteristics	70.6% had fair knowledge; 17.6% good and 11.8% low knowledg	Limited nutritional knowledge among dialysis nurses	General or Basic Nutritional

												e; higher education was associated with better knowledge		
**Sandlin et al., 2013 [37]**	Australia	Pre–post descriptive intervention study	One metropolitan HD center	HD patients	70	ESRD; HD	Assess nurse-led education on phosphate binder adherence and knowledge	Individualized nurse-led education on phosphate binders and diet	Not directly assessed; educational nursing role	Pre/post phosphate-binder knowledge survey; biochemical measures	Medication adherence, phosphate-binder knowledge, CKD-MBD markers	Correct phosphate-binder use increased from 44% to 72%; knowledge improved; biochemical markers did not significantly change	Baseline knowledge and adherence to phosphate binders were suboptimal	Educational and communicative
**Shi et al., 2013 [30]**	China	RCT	HD units of two hospitals	Adult HD patients with hyperphosphataemia	80	ESRD; long-term HD	Evaluate nurse-led intensive education for hyperphosphataemia management and phosphate knowledge	Individualized and group nurse-led education on diet and phosphate binders	Not directly assessed; educational nursing role	Knowledge questionnaire; biochemical measures	Phosphate knowledge, serum phosphorus, Ca×P, calcium, PTH, albumin	Reduced serum phosphorus and Ca×P; improved knowledge; effects sustained to 6 months	Need for individualized and sustained education on phosphate management	Educational and communicative
**Sulistyaningsih et al., 2021 [31]**	Indonesia	Qualitative phenomenological study	HD unit of Sultan Agung Islamic Hospital	HD nurses	8	ESRD; HD	Explore nurses' experiences as educators in improving diet/fluid adherence	Education, motivation, interaction, and adherence support	Perceived educator role and professional development needs	In-depth interviews	Themes describing educator role, barriers, strategies, and development needs	Five themes identified, including intensive interaction, varied educational methods, barriers,	Workload and educational barriers; need for continued professional development	Guidance and counseling

												and need to improve nurses' abilities		
**Wong et al., 2010 [32]**	Hong Kong	RCT	Renal/CAPD care setting	CAPD patients	94	ESRD; CAPD	Evaluate a nurse-led disease management program on adherence and patient outcomes	Nurse-led disease management: renal nurses as case managers (initial/final follow-up); general nurses providing intermediate follow-up	Not directly assessed; disease-management and counseling role	Disease-management outcome measures assessing adherence, QoL, symptoms, satisfaction, and service use	Diet/CAPD adherence, QoL, symptoms, satisfaction, complication control, service utilization	Improved diet and CAPD adherence, selected QoL/symptom outcomes, and satisfaction	Need for sustained, coordinated support to improve adherence	Collaborative and professional; Guidance and counseling
**Xia et al., 2016 [38]**	New Zealand	Prospective validation study	Renal ward in a metropolitan tertiary hospital	Renal inpatients	122	63 HD, 30 PD, 19 non-dialysis CKD, 3 AKI, 5 post-transplant, 2 nephrotic syndrome	Develop and validate the R-NST for undernutrition risk	R-NST validation by researchers; feasibility of nurse-completed nutritional screening assessed	Not directly assessed; nutritional screening role	R-NST and 7-point SGA	Undernutrition risk, diagnostic accuracy, dietetic referral, nurse screening compliance	R-NST showed high sensitivity and specificity; nurse screening compliance was only 22.6%	Low nursing compliance with routine nutritional screening	Clinical and advanced

Abbreviations: RCT = randomized controlled trial**;** AKI, acute kidney injury; BP, blood pressure; CAPD, continuous ambulatory peritoneal dialysis; Ca×P, calcium–phosphorus product; CKD, chronic kidney disease; CKD-MBD, chronic kidney disease–mineral and bone disorder; CKDKAT-N/CKDKATeN, Chronic Kidney Disease Knowledge Assessment Tool for Nutrition; DDFQ, Dialysis Diet and Fluid Non-Adherence Questionnaire; EQ-5D-5L, EuroQol 5-Dimension 5-Level; ESRD/ESKD, end-stage renal disease/end-stage kidney disease; HD, hemodialysis; IDWG, interdialytic weight gain; K+, potassium; NECN-ESRD, Nursing Education and Competencies in Nutrition–End-Stage Renal Disease; PD, peritoneal dialysis; PTH, parathyroid hormone; QoL, quality of life; R-NST, Renal Nutrition Screening Tool; SGA, Subjective Global Assessment.

Note: Studies are identified by author, year, and reference number; full bibliographic citations are provided in the reference list of the main manuscript.

S4. PRISMA 2020 for Abstracts Checklist (Adapted for Scoping Reviews)

Section and Topic	Item #	Checklist item	Reported (Yes/No)
**TITLE**			
Title	1	Identify the report as a scoping review.	Yes
**BACKGROUND**			
Objectives	2	Provide an explicit statement of the main objective(s) or question(s) the review addresses.	Yes
**METHODS**			
Eligibility criteria	3	Specify the inclusion and exclusion criteria for the review.	Yes
Information sources	4	Specify the information sources (e.g. databases, registers) used to identify studies and the date when each was last searched.	Yes
Risk of bias	5	Specify the methods used to assess risk of bias in the included studies.	N/A – No formal critical appraisal or risk-of-bias assessment was undertaken.
Synthesis of results	6	Specify the methods used to present and synthesise results.	Yes
**RESULTS**			
Included studies	7	Give the total number of included studies and participants and summarise relevant characteristics of studies.	Yes
Synthesis of results	8	Present results for main outcomes, preferably indicating the number of included studies and participants for each. If meta-analysis was done, report the summary estimate and confidence/credible interval. If comparing groups, indicate the direction of the effect (i.e. which group is favoured).	Yes
**DISCUSSION**			
Limitations of evidence	9	Provide a brief summary of the limitations of the evidence included in the review (e.g. study risk of bias, inconsistency and imprecision).	Yes
Interpretation	10	Provide a general interpretation of the results and important implications.	Yes
**OTHER**			
Funding	11	Specify the primary source of funding for the review.	Yes
Registration	12	Provide the register name and registration number.	Yes

Note: This checklist was adapted from the PRISMA 2020 for Abstracts checklist to reflect the methodological characteristics of a scoping review. Non-applicable requirements were identified as N/A. Aggregate participant counts were not reported due to the heterogeneity of the included populations, and meta-analytic estimates were not applicable.
